# Mega-Events, Air Pollution and Health Outcomes: A Systematic Review

**DOI:** 10.3390/antiox15050627

**Published:** 2026-05-14

**Authors:** Hiba El Khattaby, Marco Panizzolo, Federica Ghelli, Samar El Sherbiny, Valeria Bellisario, Nicoletta Colombi, Roberto Bono, Giulia Squillacioti

**Affiliations:** 1 Department of Public Health and Pediatrics, University of Turin, 10126 Turin, Italy; hiba.elkhattaby@unito.it (H.E.K.); marco.panizzolo@unito.it (M.P.); federica.ghelli@unito.it (F.G.); samar.elsherbiny@unito.it (S.E.S.); valeria.bellisario@unito.it (V.B.); giulia.squillacioti@unito.it (G.S.); 2 Biblioteca Federata di Medicina Ferdinando Rossi, University of Turin, 10126 Turin, Italy; nicoletta.colombi@unito.it

**Keywords:** air pollution, mega-events, biomarkers, mortality, hospitalization, public health, Olympics, festivals, emission control measures, oxidative stress

## Abstract

Air pollution represents a public health threat; it is co-responsible for millions of premature deaths annually and economic losses. Mega-events create abrupt changes in air pollution providing quasi-experimental settings to investigate related health impacts. This systematic review synthesizes the evidence on air pollution level changes during mega-events and associated short-term health effects, including mortality, hospitalizations and early biological responses. A literature search was conducted in PubMed, Embase, Scopus and Web of Science up to 7 April 2025. Study quality was evaluated using the EPHPP Quality Assessment Tool for Quantitative Studies. Thirty-one studies met the inclusion criteria. Mega-events without effective air pollution control measures were associated with increased pollutant levels and higher risks of respiratory and cardiovascular morbidity. Biomarker studies demonstrated rapid and reversible changes in oxidative stress and inflammatory biomarkers in response to short-term variations in air pollution. Instead, significant reductions in air pollution during mega-events were observed upon emission control measures. The evidence is predominantly from Asian countries, no Europe/Africa studies and only one from North America, limiting generalizability. Findings indicate that mega-events may influence air quality which affects human health, reinforcing the value of temporary emission control measures strategies for future mega-events. The systematic review was registered with Prospero (CRD420251032553).

## 1. Introduction

Air pollution is one of the most serious environmental health concerns worldwide, particularly in urban and densely populated areas where it causes strong negative impacts on public health [1]. When present in the atmosphere at high concentrations, air pollutants can have harmful effects on human health. The most common ones are ozone (O_3_), fine particulate matter (PM_2.5_, particulate matter with an aerodynamic diameter of 2.5 µm or less), sulphur dioxide (SO_2_) and nitrogen oxides (NOx) which originate mainly from traffic, industrial activities and energy production [2]. According to the World Health Organization (WHO) [3], the main pathway of exposure to air pollution is through the respiratory tract. Breathing in these pollutants, leads to inflammation and oxidative stress throughout the body, primarily impacting the lungs, heart and brain and ultimately increasing the risk of all-cause mortality, as well as several respiratory and cardiovascular diseases [4,5]. Consistent epidemiological evidence has linked exposure to ambient air pollution with elevated rates of chronic obstructive pulmonary disease, asthma exacerbations, ischemic heart disease, stroke and heart failure, underscoring its central role in both respiratory and cardiovascular morbidity and mortality [6,7].

The health effects of air pollution depend on both the intensity and duration. Long-term exposure has been linked to chronic heart and lung diseases, metabolic disturbances and sustained oxidative stress. [8] While short-term exposure to pollutants such as PM_2.5_, O_3_ and NO_2_ (Nitrogen dioxide) has been linked to higher rates of all-cause mortality and acute cardiorespiratory events [9]. From a mechanistic perspective, systemic inflammation and oxidative stress are key biological pathways behind both acute and chronic effects. Inhaled pollutants generate reactive oxygen species (ROS) directly or through activation of pulmonary immune cells, causing local airway inflammation and systemic inflammatory signal [10]. Biomarkers such as chemokines, exhaled nitric oxide (FeNo), nitrate/nitrite, 8-hydroxy-2′-deoxyguanosine (8-OHdG), and 8-isoprostane reflect these responses [11]. Chronic activation of inflammatory pathways can lead to tissue damage and disease progression [12], while excess ROS disrupts redox-sensitive signaling cascades, including the Nuclear factor erythroid 2-related factor 2/Antioxidant Response Element (Nrf2/ARE) pathway which regulates cellular defense mechanisms [13]. Early changes in inflammatory cytokines, oxidative stress biomarkers, endothelial function, heart rate and blood pressure can appear soon after exposure and preceding clinical events, providing a biologically plausible link between short-term exposure and acute health outcomes [14,15].

Mega-events represent a distinct and underexplored source of short-term changes in urban air pollution. Mega-events have been defined as “one-off events of a fixed duration that attract a large number of visitors, have a large mediated reach, come with large costs, and have large social and environmental impacts” [16]. Examples include international sporting competitions, political summits and large traditional festivals. Such events are characterized by abrupt changes in population density, mobility patterns, energy demand and construction activity [17]. As a result, they can substantially alter emission profiles over short periods, either worsening air quality through increased traffic and infrastructure-related emissions or improving it through temporary control measures designed to limit environmental and health impacts [18]. For instance, Chen et al. (2013) [19] used the 2008 Beijing Olympic Games to study the effect of strict temporary pollution controls and showed that these measures improved the Air Pollution Index during and shortly after the event, although most benefits had disappeared by October 2009. Overall, mega-events provide a unique opportunity to study short-term changes in air pollution and their immediate health consequences.

Despite a growing number of studies using mega-events as natural experiments in environmental and health research, the evidence remains fragmented across disciplines, pollutants, health outcomes and study designs. [18,20] In particular, there has been limited synthesis of studies simultaneously examining changes in air pollution and corresponding short-term health outcomes before, during and after mega-events. Addressing this gap is thus crucial to understand whether and under which conditions, mega-events can amplify or mitigate acute health risks through air pollution pathways. Prior systematic reviews have predominantly focused on the long-term health impacts of long-term exposure to air pollution [21,22]. For example, Boogaard et al. (2022) [23] reported moderate to high evidence that long-term exposure is associated with increased all-cause, cardiovascular, ischemic heart disease and lung cancer mortality, as well as incident asthma and acute lower respiratory infections. Overall, the review reinforces evidence linking long-term air pollution exposure to multiple adverse health outcomes. Similarly, Yazdi et al. (2022) [24] found that long-term exposure to air pollutants, particularly PM_2.5_ and O_3_ is associated with increased cardiovascular and respiratory hospital admissions among older adults, while NO_2_ show more mixed results. Likewise, Al Ahad et al. (2024) [25] observed that long-term exposure to ambient air pollution is associated with higher all-cause and cause-specific hospitalizations, including respiratory, cardiovascular, infectious and mental health conditions, with stronger effects for cumulative exposure over time. These reviews emphasize the substantial burden of long-term exposure and suggest that sustained air quality improvements can reduce healthcare use.

Short-term studies have also shown that temporary increases in pollution can trigger acute health issues as well. Liu et al. (2022) [9] reported that short-term increases in PM_2.5_ and to a lesser extent, NO_2_ and O_3_ are associated with higher all-cause mortality in the United States, with detectable effects even at concentrations below current air quality guidelines. Rakshit et al. (2025) [26] in a systematic review of short- and long-term health effects of high Air Quality Index (AQI) exposure, summarized evidence that short-term increases in air pollution are linked to acute respiratory and other multisystem morbidities. Seihei et al. (2024) [27] highlighted that Andimeshk city experiences very high particulate matter levels, with PM where particles are less than 10 micrometres in diameter (PM_10_) often exceeding legal limits due to dust storms and regional dust transport, thereby exposing the population to substantial short-term health risks. In line with these findings, Zhang et al. (2025) [28] reported that short-term exposure to PM_2.5_, PM_10_, SO_2_, NO_2_ and Carbon Monoxide (CO) during peak traffic hours is associated with increased hospitalizations for respiratory diseases, with particularly pronounced effects among children and older adults. However, only a small part of this literature has examined the acute health effects of short-term air pollution exposure during mega-events, where pollution levels may vary significantly due to traffic limitations, construction activities or temporary emission control policies.

Therefore, this study aims to systematically review the evidence on the effects of mega-events on air pollution and related health outcomes. Specifically, it examines changes in air pollutant concentrations and health indicators including hospitalizations, mortality and biological markers before, during and after mega-events, and compares these effects across different event types and contexts. By focusing on acute exposure windows, this review seeks to clarify the immediate health implications of mega-event-related air pollution and to inform future public health and urban planning strategies.

## 2. Materials and Methods

The present systematic review protocol is registered on PROSPERO database, last check made on 14 April 2025 and publish on the 9 April 2026, ID: CRD420251032553 Available from: https://www.crd.york.ac.uk/PROSPERO/view/CRD420251032553 (accessed on 9 April 2026). And the systematic review is reported following the PRISMA 2020 statement guidelines.

### 2.1. Study Selection

Eligible articles were searched and identified in PubMed, Embase, Scopus and Web of Science (WoS) up to 7 April 2025. The search string aimed to identify original research articles assessing the impact of air pollution associated with mega-events on human health outcomes including the following terms: ‘Air Pollution’, ‘Air Pollutants’, ‘mega-events’, ‘Olympics’, ‘Festivals’, ‘Health’, ‘Respiratory Diseases’, ‘Cardiorespiratory Diseases’, ‘Hospitalisations’, ‘Mortality’. Full strings are reported in Appendix A.

### 2.2. Inclusion and Exclusion Criteria

Original observational or interventional studies conducted in urban or suburban areas during mega-events (sports, cultural, festival or political gatherings) assessing air pollution levels (e.g., PM_2.5_, NO_2_, O_3_, CO, etc.) and their effects on human health outcomes were considered potentially eligible. The populations under investigation include general or specific subgroups, with outcomes such as respiratory, cardiovascular, mortality, hospitalization or early biological responses such as oxidative stress and inflammation. Comparative studies evaluating exposure before, during and after the events were also included. On the contrary, we excluded editorials, opinion papers, conference abstracts, protocols, dissertations, reviews, meta-analyses and studies that did not measure air pollution during a mega-event or that did not assess health outcomes. Two reviewers completed the article selection in a blind process, starting from the screening of titles and abstracts according to the inclusion and exclusion criteria declared. Included resources underwent a second screening phase based on full texts evaluation using Rayyan (http://rayyan.qcri.org). Disagreements on article selection were discussed and eventually submitted to a third reviewer.

### 2.3. Data Extraction

Data extraction was independently performed by two researchers using structured spreadsheets. For studies on mortality and hospitalizations, we collected information on author, year, title, country, study design, population characteristics, sample size, age, sex, type and measurement of air pollution, concentrations (before, during and after the mega-event), type and year of mega-event, observation period, outcomes assessed (mortality and hospitalizations) and outcomes quantification at each time point [counts, mean ± Standard Deviation (SD), median (Interquartile Range, IQR)] or mean with 95% Confidence Intervals, CIs) along with statistical analysis details and main results. For biomarker biomonitoring studies, extraction included similar variables with additional details on biomarker type, biological matrix, unit of measure and assessment methods. Outcomes were measured before, during and after the mega-event were extracted using the same metrics. The timing of the pre-event and post-event periods was recorded as defined by each original study, because these event windows differed across studies. Because of this methodological heterogeneity, we synthesized the findings narratively rather than applying a single uniform sampling window across all studies.

### 2.4. Quality Assessment

The quality assessment of the included articles was performed by two independent reviewers using the Effective Public Health Practice Project (EPHPP) Quality Assessment Tool for Quantitative studies available at https://www.ephpp.ca/quality-assessment-tool-for-quantitative-studies/ (accessed on 3 February 2026). This instrument evaluates risk of bias across eight methodological categories—selection bias, study design, confounders, blinding, data collection methods, withdrawals and dropouts, intervention integrity, and analysis—by integrating objective criteria with the judgments of experts who meet predefined qualifications. An overall quality score is then calculated by summing the domain ratings, yielding a numeric value that determines the final classification: scores ≤ 5 are designated weak, scores 6–7 are moderate, and scores ≥ 8 are considered strong. This systematic approach ensures that the risk of bias is transparently assessed across all included studies and any discrepancy between reviewers was discussed, and, if required, a third reviewer was consulted.

## 3. Results

### 3.1. Qualitative Synthesis

**Figure 1:**
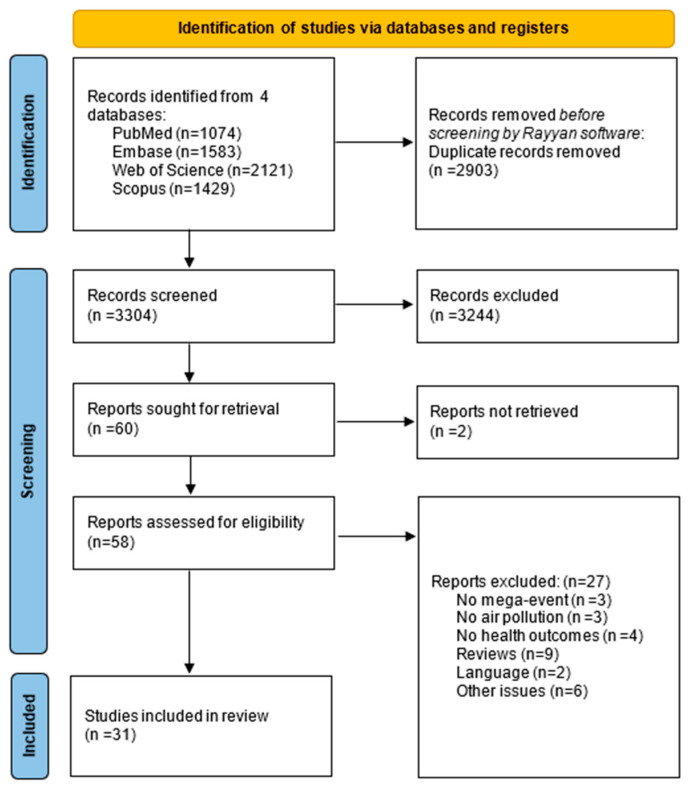
Flowchart of the identification for eligible studies from a search among original art adapted from Page et al., 2021 [29].

As described in Figure 1, among the 6207 items initially identified, 2903 duplicates were removed before screening. Titles and abstracts of the remaining 3304 were screened, 58 full texts were assessed and the exclusion criteria lead to the removal of 27 articles. A number of 31 research articles were finally included in the present systematic review. Among the excluded articles, three did not involve any mega-event, three did not assess air pollution, and four did not report relevant health outcomes (e.g., mortality, hospitalization and early biological responses). Additionally, nine review papers, two articles written in languages other than English and six studies with other methodological issues were excluded. The procedure is summarized by the PRISMA diagram reported in Figure 1.

### 3.2. Study Setting and Mega-Events Characteristics

As shown in Table 1, a total of 31 studies were included in this review, comprising mainly observational designs. The studies were published between 2010 and 2024 with the vast majority conducted in China (*n* = 28), followed by India (*n* = 2) and the USA (*n* = 1). Most studies assessed the total general population during mega-events and in a subset of studies where a specific sample was reported the number of participants ranged from 11 to 788 individuals. The included studies were mainly observational and varied substantially in sample population characteristics, observation period and mega-event context, which limits the direct comparison of outcome estimates across studies, as summarized in Table 1. The mega-events were mainly sport-related with the Beijing Olympics and Paralympic games 2008 being the most frequently studied event (*n* = 22), followed by the Asian Games (*n* = 4). Other sport events included the Nanjing Youth Olympics Games 2014 (*n* = 1), the World Military Games 2019 (*n* = 1) and the Atlanta Olympics Games 1996 (*n* = 1). Cultural and religious festivals were also represented including Diwali, Dusshera, Deepawali and Holi festivals in India (*n* = 2). The observation period varied across studies, with mega-event durations ranging from 2 to 51 days and an average duration of approximately 31 days (±10.2 days).

### 3.3. Mega-Event-Related Air Pollution, Mortality and Hospitalization

Table 2 provides an overview of the characteristics and main findings of the studies that investigated the relationship between changes in air pollution during mega-events and mortality and hospitalization outcomes. A total of 14 studies were included [30,32,33,34,35,36,39,41,44,45,48,56,57,59], all of which measured ambient air pollutant concentrations before, during, and after the mega-event using data obtained from official environmental monitoring networks. The pollutants most frequently examined were PM_2.5_, SO_2_, NO_2_, O_3_, SO_2_, NO and particle number concentration (PNC) [32,45], measured through validated monitoring instruments such as tapered element oscillating microbalance (TEOM) systems [32,45], chemiluminescence analyzer [44], fluorescence-based monitors and particle mobility spectrometers [39,57]. Health outcomes were extracted from mortality registries and hospital admission databases and mainly comprised all-cause mortality (*n* = 2) ([35,41]), cardiovascular and respiratory mortality (*n* = 5) ([33,34,45,47,56]), hospitalization (*n* = 4) ([32,36,39,44]), health risks (*n* = 2) ([30,57]) and emergency department visits (*n* = 1) ([59]). Most studies used time-series designs and applied statistical models such as generalized additive models (GAM), quasi-Poisson regression or linear regression, with adjustments for meteorological variables, long-term temporal trends and day-of-week effects to estimate the short-term impact of pollutant fluctuations on health outcomes. Regarding the results, as shown in Table 2, among the 14 included studies [30,32,33,34,35,36,39,41,44,45,48,56,57,59], mortality outcomes were mainly examined in relation to short-term variations in PM_2.5_, PM_10_, NO_2_ and O_3_ during mega-events or pollution control periods. In most studies ([34,41,45,48]), reductions in particulate matter during intervention periods were associated with decreases in all-cause, cardiovascular and respiratory mortality. He et al. (2016) [41] reported that a 10 µg/m^3^ decrease in PM_10_ was associated with an 8.4–9.6% reduction in all-cause mortality and an 8.8% reduction in cardiovascular mortality. Similarly, Lin et al. (2014) [48] reported reductions in daily mortality during the Asian Games period, with all-cause mortality, Mean(SD), decreasing from 32 (7) to 25 (5) per day, cardiovascular mortality from 11 (4) to 8 (3) per day, and respiratory mortality from 6 (3) to 5 (2) per day, coinciding with lower PM_10_ concentrations. During the Beijing Olympic and Paralympic Games, Su et al. (2015) [45] reported that reductions in air pollution, particularly in PNC, were associated with a decrease in cardiovascular mortality risk during the period of strict emission control measures. Whereas, Zhang et al. (2019) [34] reported a reduction in stroke mortality during the 2010 Guangzhou Asian Games following the implementation of an air pollution control program. Daily stroke mortality decreased during the Asian Games (−18.26%). Reductions were also observed for ischemic stroke (−12%) and hemorrhagic stroke (−25.75%). PM_2.5_, SO_2_, and NO_2_ were positively associated with stroke mortality counts, while PM_10_ levels significantly decreased during the Asian Games period. By contrast, studies ([33,35,56]) conducted during periods of increased air pollution reported higher risks. Wu et al. (2019) [33] reported that a 10 µg/m^3^ increase in NO_2_ was associated with a 1.89% increase in total mortality (95% CI 1.49–2.29), while a 10 µg/m^3^ increase in O_3_ was associated with a 0.60% increase in total mortality (95% CI: 0.47–0.74). In addition, IQR increases in O_3_ were associated with higher cardiorespiratory mortality and a 4.42% increase in cardiovascular mortality (95% CI: 3.16–5.69). Zhang et al. (2011) [56] reported stronger associations between PM_10_ and cardiovascular mortality, whereas NO_2_ showed stronger effects on respiratory mortality. Sinha et al. (2019) [35] similarly observed higher excess mortality with increased PM_2.5_ levels. For hospitalizations and emergency department visits, Peel et al. (2010) [59] reported 2% to 4% increases in respiratory emergency visits associated with O_3_, while particulate matter and gaseous pollutants were linked to cardiovascular visits. Gupta et al. (2019) [39] observed that respiratory hospital admissions during the Holi festival were approximately twice as high as before the event and 1.3 times higher than after Holi. PM_10_ showed a stronger impact than PM_2.5,_ particularly for upper respiratory admissions. Daga et al. (2019) [36] also reported increases in total, respiratory, cardiac and stroke admissions following post-Diwali rises in PM_2.5_ and PM_10_. During the Olympic emission control period, Su et al. (2016) [44] reported higher cardiovascular emergency room visits during the non-Olympic period when air pollution levels were higher, while lower pollution levels during the Olympic period were associated with reduced cardiovascular Emergency Room Visits (ERV). PM_2.5_ showed adverse effects on cardiovascular ERV, with an interquartile range increase (68 µg/m^3^) associated with a relative risk of 1.022 (95% CI: 0.990–1.057), particularly among females, although this association did not reach statistical significance because the confidence interval crossed 1.0. Breitner et al. (2021) [32] observed lower respiratory and pneumonia mortality during the Olympic intervention period enforced under strict emission control measures, the associations were mainly observed for particle number concentrations, with an increase of 7958 particles/cm^3^ in ultrafine particles associated with a 16.3% increase in respiratory mortality.

**Figure 2:**
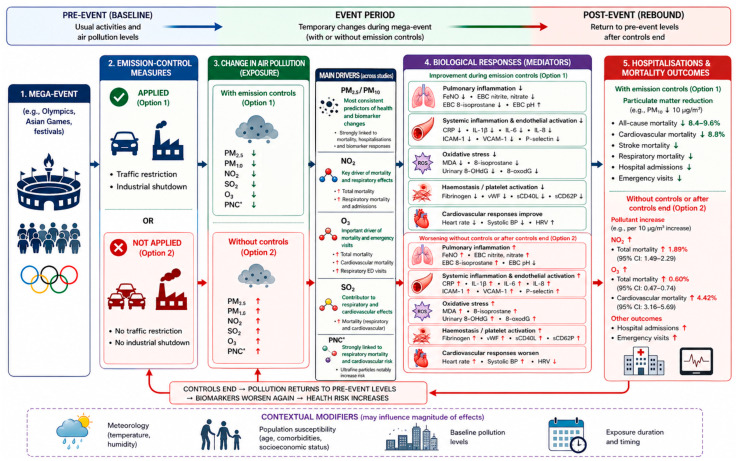
Conceptual model summarizing the relationship between mega-event-related emission-control measures, short-term air-pollution changes, early biological responses and hospitalization/mortality outcomes. Note: Conceptual model based on 31 studies of mega-events: 14 studies assessed hospitalization and/or mortality outcomes, 17 studies assessed early biological responses. **Abbreviation**: ↑: increase, ↓: decrease, PM, particulate matter; NO_2_, nitrogen dioxide; O_2_, ozone; SO_2_, sulfur dioxide; PNC, particle number concentration; FeNO, fractional exhaled nitric oxide; EBC, exhaled breath condensate; CRP, C-reactive protein; IL, interleukin; ICAM-1, intercellular adhesion molecule-1; VCAM-1, vascular cell adhesion molecule-1; vWF, von Willebrand factor; sCD40L, soluble CD40 ligand; sCD62P, soluble P-selectin; MDA, malondialdehyde; 8-OHdG, 8-hydroxy-2′-deoxyguanosine; HRV, heart rate variability; BP, blood pressure.

As illustrated in Figure 2, mega-events can temporarily alter air pollution exposure, with emission controls generally reducing particulate pollution and associated mortality and hospitalizations outcomes, whereas the absence of emission controls is linked to increases in pollutant levels and adverse health impacts.

Overall, the mortality and hospitalization studies showed a consistent pattern in both effect size and statistical significance across event types, pollutants and outcome definitions. Studies conducted before–during–after mega-events with stricter temporary emission controls generally showed cleaner reductions in PM_2.5_, PM_10_ and NO_2_ and were more often associated with lower mortality or hospitalization risks, whereas festival-related studies without emission controls more often showed the opposite pattern. These differences likely reflect how strong the pollution controls were, how much air pollution changes, who was studied and how each study was designed.

### 3.4. Mega-Event-Related Air Pollution and Early Biological Responses

Table 3 presents the characteristics and main findings of studies observing early biological responses to variations in air pollution during mega-events, with a specific focus on inflammatory and oxidative stress biomarkers. Across 17 studies ([31,37,38,40,42,43,46,47,49,50,51,52,53,54,55,58,60]), ambient air pollutant concentrations were measured before, during and after mega-events using standardized environmental monitoring systems, and biological samples were collected in parallel to assess short-term physiological responses to exposure changes. The pollutants most frequently monitored included PM_2.5_, PM_10_, O_3_, NO_2_ and particle number concentration. Biological assessments were conducted using multiple matrices, including blood ([31,37,40,42,43,47,50]), urine ([43,46,47,50,52]), saliva and exhaled breath condensate ([49,50,52,53,54]). The biomarkers investigated mainly reflected systemic inflammation, oxidative stress and autonomic or cardiovascular responses, such as C-reactive protein (CRP), interleukins (IL-6, IL-8), tumor necrosis factor (TNF-α), malondialdehyde (MDA), 8-hydroxy-2′-deoxyguanosine (8-OHdG) and heart rate variability (HRV). Most studies adopted repeated measure or panel designs and applied linear mixed models, generalized linear models or time-series analyses to estimate short-term associations between pollutant variations and biomarker fluctuations, accounting for meteorological and temporal confounders.

Regarding the results, as shown in Table 3, among the 17 studies ([31,37,38,40,42,43,46,47,49,50,51,52,53,54,55,58,60]), reductions in air pollution during intervention periods were generally associated with decreases in inflammatory, oxidative stress and cardiovascular biomarkers. For example, Li et al. (2019) [37] reported lower levels of inflammatory chemokines, including Regulated on Activation, Normal T Cell Expressed and Secreted (RANTES), Monocyte Chemotactic Protein-2 (MCP-2) and Thymus and Activation-Regulated Chemokine (TARC) during the 2008 Beijing Olympic period with the strict emission control, while increases in air pollution after the Games were associated with higher levels of these biomarkers and positive associations with IL-8, Eotaxin-1 and Growth-Regulated Oncogene Alpha (GRO-α). Similarly, Li et al. (2017) [42] reported significant changes in inflammatory biomarkers across the before, during, and after periods of the 2014 Nanjing Youth Olympic Games, including soluble CD40 ligand (sCD40L), Interleukin-1 beta (IL-1β), CRP and Vascular Cell Adhesion Molecule (VCAM-1). Higher PM_2.5_ and O_3_ concentrations were associated with increased serum levels of sCD40L, Intercellular Adhesion Molecule 1 (ICAM-1), VCAM-1, P-selectin and IL-1β, while reductions in PM_2.5_ and O_3_ were associated with decreases in inflammatory biomarkers. Taken together, these two Olympics studies show a similar short-term biological response to air pollution changes but the specific biomarkers and exposure responses patterns were not identical, suggesting that the effect depends on the event setting, pollutant mixture and biomarker type.

Similar patterns were reported for pulmonary and systemic inflammation markers. For instance, Huang et al. (2012) [54] reported that reductions in air pollution during the 2008 Beijing Olympic control period were associated with decreases in FeNO and oxidative stress biomarkers, indicating reduced pulmonary inflammation. In contrast, higher air pollution levels in the 0–48 h were associated with increases in FeNO, Exhaled Breath Condensate (EBC) nitrite and nitrate and decreases in EBC pH, while pollution exposure 48–96 h before measurement was associated with increases in EBC 8-isoprostane and markers of DNA oxidative damage. Similarly, Zhang et al. (2013) [52] reported that during the 2008 Beijing Olympic period, when air pollution decreased, inflammatory, oxidative stress and hemostasis biomarkers were reduced, accompanied by improved cardiovascular physiology. After the Olympics, when pollution levels increased again, increases were observed in pulmonary inflammation markers (FeNO, EBC nitrite, nitrate, nitrite+nitrate, pH, 8-isoprostane), systemic inflammation and oxidative stress markers (fibrinogen, von Willebrand Factor (vWF), urinary 8-OHdG), as well as hemostasis and platelet activation markers (sCD40L, sCD62P, vWF), together with increases in cardiovascular measures such as heart rate and systolic blood pressure. Lin et al. (2015) [47] reported that higher levels of Black carbon (BC), PM_2.5_, SO_2_, NO_2_ and CO were associated with increases in malondialdehyde and 8-oxodG, while Gong et al. (2013) [53] found that reductions in air pollution during Olympic control measures were associated with decreases in EBC MDA, with levels increasing again after the controls ended. Studies examining cardiovascular and hemostasis biomarkers also reported similar patterns. Rich et al. (2012) [55] observed decreases in fibrinogen, vWF, soluble P-selectin (sCD62P), sCD40L and heart rate during the Beijing Olympic Games when particulate and gaseous pollutants decreased by 13–60%, whereas these biomarkers increased again after pollutant concentrations returned to pre-Olympic levels. In addition, changes in air pollution were associated with physiological responses, including increases in blood pressure with higher PM_2.5_ exposure, in the same study during the Seventh World Military Games, no significant response was observed for Glutathione Reductase (GR) despite changes in other oxidative stress and inflammatory biomarkers [31] and altered heart rate variability in individuals exposed to high traffic-related particulate matter levels [60]. Biomarkers of exposure also responded to pollution changes, for example, Gong et al. (2015) [46] reported decreases in urinary metabolites of traffic-related pollutants during the Olympic period, followed by increases after the Games.

Figure 2 shows that emission-control periods were linked to improved early biological responses, with lower levels of inflammation, oxidative stress, respiratory and cardiovascular biomarkers, whereas these markers worsened after the controls were lifted.

Overall, Table 3 shows that short-term reductions in air pollution during mega-events were often accompanied by improvements in early biological responses, particularly inflammatory, oxidative stress, hemostatic and cardio-respiratory biomarkers. Across studies, PM_2.5_ and PM_10_ were the pollutants most frequently associated with changes in biomarkers such as CRP, IL-1β, IL-6, IL8, VCAM-1, MDA, 8-isoprostane and 8-OHdG, while traffic and combustion- related pollutants including NO_2_, SO_2_, BC and CO showed similar but more variable associations. Several studies also reported that biomarker levels worsened again after pollution returned to pre-event levels indicating a consistent short-term biological response to changes in air quality.

### 3.5. Risk of Bias Assessment

All included studies were assessed with the EPHPP Quality Assessment Tool for Quantitative Studies according to their respective designs. Overall, 77.42% of the studies were rated as ‘weak’, 22.58% were rated as ‘Moderate ‘quality and no study received a strong rating. Most of the weak ratings reflected limitations in the study design and confounding control, and because the included studies used different designs and event windows, the relation between study quality and findings was examined qualitatively rather than through a pooled statistical comparison. Data extraction was performed systematically according to predefined criteria to ensure consistency across all studies. (Supplementary Materials: Table S1).

## 4. Discussion

This review shows that mega-events provide a unique opportunity to observe how rapid changes in air pollution are associated with short-term changes in mortality, hospitalizations and early biological responses. Mega-events can generate abrupt and large-scale shifts in urban emissions.

Preparatory construction activities and increased mobility may temporarily worsen air quality, whereas the implementation of traffic restrictions, industrial shutdowns, and other emission control measures can substantially reduce pollutant levels over short periods [61]. Mega-events are not homogeneous and their health effects vary according to the nature of the event and the associated emission profile. Evidence from several Olympic and Asian Games interventions showed marked reductions in pollutants such as PM_2.5_, PM_10_, NO_2_, SO_2_ and particle number concentrations, while festivals without emission controls, such as Diwali or Holi, were associated with sharp pollution increases. Sporting events are therefore more often linked to temporary emission-control measures and pollutant reductions, whereas festivals and religious gatherings may generate pollution increases because of fireworks, biomass burning, crowding and transport-related emissions. Accordingly, the direction and magnitude of health effects may differ across event types and this should be considered in the interpretation of the evidence.

In this review, several studies reported that strict emission controls during mega-events reduced ambient concentrations of PM_2.5_, PM_10_, NO_2_, SO_2_ and other pollutants which were followed by decreases in all-cause, cardiovascular and respiratory mortality. [32,34,41,45,48]. These findings are biologically plausible, given that inhaled particulate matter and traffic-related gases are known to trigger pulmonary oxidative stress and inflammation that rapidly propagate systemically, promoting endothelial dysfunction, autonomic imbalance and a pro-thrombotic state that can precipitate acute cardiovascular and respiratory outcomes [62,63,64].

### 4.1. Early Biological Responses: Oxidative Stress and Inflammation

Across the included early biological responses study, the pattern was not identical for all pollutants. PM_2.5_ and PM_10_ were the pollutants most consistently associated with changes in inflammatory, oxidative stress, hemostatic and cardio-respiratory biomarkers, whereas NO_2_, SO_2_, BC and CO showed similar but more variable associations.

Experimental and clinical studies show that PM and PM_2.5_ deposit deeply in the lungs, enter cells and accumulate in mitochondria, where they disrupt the electron transport chain and produce ROS, leading to oxidative stress, mitochondrial DNA damage, cytochrome-c release and apoptosis [65,66]. This oxidative stress activates redox-sensitive signaling pathways (e.g.,Mitogen-Activated Protein Kinase (MAPK), Nuclear Factor kappa-light-chain-enhancer of activated B cells (NF-kβ), Activator Protein-1 (AP-1)), which up-regulate pro-inflammatory cytokines and adhesion molecules, promoting systemic inflammation, endothelial dysfunction, vasoconstriction and platelet activation [67]. These pathways provide a mechanistic potential explanation for the increases in mortality and hospital admissions observed during periods of higher pollution reported in this review. In addition, controlled exposure studies to urban PM and diesel exhaust have shown reduced vasodilatory responses and decreased heart-rate variability, effects that are attenuated when exposure is lowered, supporting the idea that short-term reductions in pollution can rapidly reduce cardiovascular strain and acute mortality risk [63,68]. A recent multi-omics systematic review confirms that PM exposure is consistently associated with higher oxidative stress markers, altered pro-oxidant metabolites (eicosanoids, ceramides), depletion of antioxidant pathways (glutathione, vitamins C and E), and disruptions in metabolic and inflammatory processes that favor a pro-inflammatory, pro-oxidative profile, which is consistent with the biomarker increase observed in this review during emission control periods [69]. Conversely, when mega-events are associated with increased emissions (e.g., traffic congestion, fireworks, biomass burning), higher short-term concentrations of PM_2.5_, PM_10_, NO_2_ and SO_2_ are expected to intensify oxidative stress and systemic inflammation, which aligns with the increases in mortality, hospitalizations and biological markers reported in our results during high-exposure periods, and is consistent with epidemiological evidence showing that even modest day-to-day increases in these pollutants are linked to higher risks of myocardial infarction, stroke, heart failure and cardiorespiratory mortality, with no clear safe threshold [70]. This interpretation is further supported by previous research supporting the notion that individuals with pre-existing respiratory disease are especially vulnerable to these acute pollutant spikes. In an Italian multicenter cohort, long-term exposure to traffic-related air pollution was associated with a markedly higher prevalence of chronic obstructive pulmonary disease (COPD) and more severe disease progression [71]. Moreover, a multipollutant analysis linking long-term exposure to a mixture of PM, NO_2_ and O_3_ with asthma and rhinitis demonstrated that patients with allergic airway conditions had amplified inflammatory responses and poorer symptom control [72]. Together, these results indicate that people with respiratory diseases or vulnerable groups suffer from the worst effects when pollution surges during mega-events.

Toxicological and in vitro studies support the evidence that intervention reducing air pollution can result in a decrease in inflammatory, oxidative stress and cardiovascular biomarkers, with a new increase in biomarker levels when pollution return to pre-event levels. Particulate matter with high oxidative potential induces ROS generation, lipid peroxidation and oxidative DNA damage disrupts endothelial tight junctions via ROS-dependent calcium influx and calpain-mediated degradation of the junctional protein ZO-1 and up-regulates adhesion molecules such as ICAM-1 and VCAM-1, particularly for particles derived from combustion and metal-rich sources, antioxidant treatment has been shown to prevent these effects, supporting a causal role of oxidative stress pathways [73,74]. These findings are consistent with broader evidence showing that airborne particulate matter induces oxidative stress and inflammation across multiple organ systems, which are central mechanisms linking air pollution to cardiovascular and respiratory disease [75]. They also align with epidemiological and biomarker data from Italian studies on vulnerable people and air pollution. In the GEIRD multi-case–control study, Squillacioti et al. (2024) [76] found that adults with asthma, COPD and other airway diseases who were exposed to higher urban air pollution levels had significantly increased systemic oxidative-stress biomarkers compared with less exposed subjects, reinforcing the central role of redox imbalance and systemic inflammation as pathways through which air pollution can exacerbate respiratory morbidity. In parallel, Schilirò et al. (2026) [77] demonstrated that PM_10_ from the Po Valley has high oxidative and toxic potential in vitro, reinforcing the idea that changes in particle load and composition during mega-events can rapidly modulate oxidative stress and related biomarker profiles possibly also in exposed populations, as observed in our review.

### 4.2. Mortality and Hospitalisations Outcomes

Across the included mortality and hospitalization studies, the pattern was not identical for all pollutants. PM_10_ appeared more consistently associated with cardiovascular mortality, NO_2_ with respiratory mortality, and O_3_ with total mortality or respiratory emergency visits, whereas PM_2.5_ and PM_10_ were the pollutants most frequently linked to biomarker changes.

Studies conducted during the Beijing 2008 Olympics showed that short-term reductions in air pollution were associated with changes in EBC markers, including pH, nitric oxide, nitrite and nitrate, demonstrating that short-term variability in PM and NO_2_ directly modulates airway oxidative and inflammatory responses in healthy adults [65], which is in agreement with the decreases in FeNO, EBC nitrite/nitrate and 8-isoprostane reported in the Olympic panel studies included in this review. Consistent with the mortality and hospitalization findings, a nationwide case-crossover study in 292 Chinese cities evaluating the Air Pollution Prevention and Control Action Plan (APPCAP) found that between 2013 and 2017 mean PM_2.5_ and CO concentrations fell by about 29% and 20%, respectively, and these declines were accompanied by a roughly 30% decrease in cause-specific hospital admissions [78], while a quasi-experimental study of Tokyo’s diesel emission control ordinance similarly showed that, compared with Osaka, larger decreases in PM_2.5_ were paralleled by greater declines in age-standardized mortality, including a 22% reduction in pulmonary disease mortality and a 4.9% reduction in lung cancer mortality over a decade, consistent with a causal benefit of targeted emission reductions on respiratory deaths [79]. Moreover, in Makkah, monthly NO_2_ and O_3_ levels were more strongly associated with cardiovascular and respiratory mortality during the crowded Hajj seasons than in non-Hajj periods, suggesting that pollution peaks superimposed on vulnerable populations can amplify death rates. [80] The fact that, during the COVID-19 years when pilgrim numbers and emissions were sharply reduced, most pollutant mortality associations in Makkah became inverse or non-significant (with the partial exception of SO_2_) further supports the interpretation that it is the concurrence of pollution peaks with a concentrated, high-risk population that drives the excess cardiorespiratory mortality observed during Hajj [80]. In addition, toxicological studies conducted during the Nanjing Youth Olympic Games demonstrated that air-quality interventions can reduce not only pollutant concentrations but also the oxidative and cytotoxic potential of particulate matter, supporting the reductions in oxidative stress biomarkers observed in this review [81]. These lines of evidence are complemented by previous research during the Torino 2006 Winter Olympic Games, where targeted emission-control measures around the competition venues were linked to measurable reductions in ambient benzene and formaldehyde concentrations [82] and to attenuated mutagenic activity of PM_2.5_ in the Po Valley [83], showing that mega-event interventions can modify both gaseous carcinogens and the genotoxic fraction of PM in a way that is consistent with the biomarker improvements observed in our review. More recently, the integration of effect-based bioassays into routine PM_10_ monitoring in the same region [77] has shown that periods with stricter air-quality measures are characterized by a lower oxidative and mutagenic potential of particulate samples, mirroring the pattern of reduced toxicological potency and improved biomarker profiles that we report during mega-events. Taken together, the studies in Turin (2006) and later monitoring in the Po Valley both support our findings, they show that when emission controls are put in place for big events, air-pollutant levels decrease, the remaining pollutants become potentially less detrimental, and we can see real improvements in health markers and actual health outcomes.

### 4.3. Methodological Consideration and Excluded Studies

There were two studies, Thakur et al.(2010) and Huang et al.(2017) [84,85] that were mainly based on simulated estimations rather than directly calculated exposure response analysis. Because they did not provide clearly quantified effect variations derived from observed data, they have not been included in the present systematic review. However, is worth mentioning their contribution to the scientific literature; the direction of their findings was aligned with studies that provided measured effect estimates, supporting the overall consistency of the observed evidence. In the case of Zhu et al. (2021) [86], the biomarker findings were primarily based on correlation analyses rather than fully quantified exposure response gradients, which limits the precision of interpretation. Nevertheless, the observed direction of change remained consistent with other biomarker studies that reported quantified reductions. Additionally, a further article by Ghada et al. (2021) [87], the only European paper we found on Oktoberfest in Munich, Germany, did not report results specifically for emergency-department visits during the Oktoberfest period but only for annual data without isolating the event period, for this reason, it could not be directly integrated into our before–during–after framework. Even though their paper still adds useful background on festival-related pollution.

### 4.4. Strength and Limitation

This review has several strengths. First, it provides a comprehensive synthesis of studies examining the relationship between air pollution changes during mega-events and health outcomes, highlighting how temporary emission control measures can influence both clinical outcomes (mortality and hospitalizations) and early biological responses. Another strength of this review is the inclusion of studies investigating not only clinical outcomes but also biomarkers related to inflammation, oxidative stress and cardiovascular function, which helps to better understand the potential biological mechanisms linking air pollution exposure to adverse health effects. However, several limitations should also be considered when interpreting the findings. The geographical distribution of the included studies was uneven. Most of the available evidence comes from Asian countries, particularly China, where several studies were conducted during the Beijing Olympic Games [32,35,37,38,40,41,43,44,45,46,47,49,50,51,56,57,58,60,85], while others examined events, such as the Asian Games [33,34,42,48] or large-scale festivals in India [36,39]. In contrast, no eligible studies were identified from Europe or Africa and only one study was conducted in North America [59]. This pattern reflects the current evidence base rather than a restriction in our search strategy and it may limit the generalizability of the findings to other regions with different environmental conditions, pollution levels and sources and population characteristics.

### 4.5. Public Health Implications and Future Perspectives

The findings of this review highlight several gaps in the current literature that might be a starting point to suggest directions for public health and future research. From a public health perspective, the consistent improvements observed during periods of strict emission control measures highlight the potential benefits of implementing similar strategies beyond mega-events such as the Olympic Games. In contrast, festivals and large public gatherings without emission controls, such as Diwali or Holi, were often associated with sharp increases in pollution and adverse health outcomes. This suggests that stricter regulation of emission sources during such events, including traffic restrictions, control of fireworks, and temporary industrial measures, could substantially reduce short-term health risks. Therefore, future mega-events planning should prioritize early targeted emission-control strategies tailored to the size and timing of the expected pollution peak.

From a research perspective, several gaps remain. First, there is a need for more studies conducted in underrepresented regions, particularly Europe, America, Australia and Africa, in order to improve the generalizability of the findings and account for differences in pollution sources and population characteristics. Second, future studies should aim to better characterize the impact of specific emission sources during mega-events, including traffic, biomass burning, and fireworks, as well as their contribution to pollutant mixtures and toxicity. There is also a need for more integrated study designs combining clinical outcomes and biological markers within the same populations, in order to strengthen the causal link between exposure, early biological responses, and health effects. Finally, longitudinal studies are needed to determine whether repeated short-term improvements in air quality can translate into sustained long-term health benefits. At the individual level, these findings suggest that personal preventive strategies may also play an important role, particularly during high-exposure events. Reducing outdoor activities during peak pollution periods and the use of personal protective equipment, such as face masks with an adequate filtering capacity, may help limit exposure, especially among vulnerable populations including older adults and individuals with pre-existing cardiovascular or respiratory conditions. Increasing public awareness of short-term pollution peaks during mega-events and promoting simple protective behaviors could further contribute to reducing health risks.

## 5. Conclusions

Air pollution, when altered abruptly during mega-events, produces immediate and measurable health effects. The studies assembled in this review demonstrate that emission control measures, such as traffic restrictions, industrial shutdowns and firework bans, implemented for events like the Olympic Games, Asian Games or large festivals consistently lowered concentrations of PM_2.5_, PM_10_, NO_2_, SO_2_ and related pollutants. These reductions were paralleled by declines in all-cause, cardiovascular and respiratory mortality, hospital admissions and early biological responses. For example, one included study reported that a 10 μg/m^3^ decrease in PM_10_ was associated with an 8.4 to 9.6% reduction in all-cause mortality and an 8.8% reduction in cardiovascular mortality [41]. In contrast, events without strict emission controls causes sharp pollution increases that coincided with rises in mortality, hospitalizations and elevated biomarker levels. These findings support strict emission controls for mega-events, such as traffic restrictions, industrial shutdowns and firework bans, especially in settings with high baseline pollution and vulnerable populations. The review also highlights several methodological gaps and the dominance of evidence from Asian contexts (e.g., Beijing Olympic studies) with less data from Europe, Africa, the Americas and Oceania, limiting the generalizability of findings.

## Figures and Tables

**Table 1:** Overview of study settings and mega-events characteristics.

Ref.	Study Design	Country	Mega-Event, Year	Sample Population (*n*)	Total Observation Period	Mega-Events Duration (Days)	Quality Assessment Score
[30]	Observational study	China	Asian Games, 2023	N/A	23 September 2023–8 October 2023	16	5
[31]	A panel study	China	VII World Military Games, 2019	70	September 2019–January 2020	10	3
[32]	Observational study	China	Beijing Olympics, 2008	12.3 million registered inhabitants	20 May 2008–1 December 2008	51	4
[33]	Observational study	China	Asian Games, 2010	14.5 million resident	12 November 2010–18 December 2010	15	6
[34]	Observational study	China	Asian Games, 2010	12.7 million permanent residents	1 January 2006–21 December 2011	15	7
[35]	Observational study	China	Beijing Olympics, 2008	N/A	30 June 2008–5 October 2008	16	6
[36]	Observational study	India	Diwali and Dusshera festivals, 2017	462 (Dussehra), 788 (Diwali)	27 September 2017–22 October 2017	6	7
[37]	A panel study	China	Beijing Olympics, 2008	104	08 August 2008–17 September 2008	16	4
[38]	A panel study	China	Beijing Olympics and Paralympic games, 2008	26	08 August 2008–17 September 2008	16 (Olympics) + 11 (Paralympics)	6
[39]	Observational study	India	Holi festival, 2018	2211 entries between pre-Holi and post-Holi	27 February 2018–3 March 2018	2	6
[40]	A panel study	China	Beijing Olympics and Paralympic games, 2008	180	8 August 2008–17 September 2008	16 (Olympics) + 11 (Paralympics)	4
[41]	Observational study	China	Beijing Olympics, 2008	200 million people (34 urban city districts)	1 November 2007–20 September 2008	16	4
[42]	A Quasi-experimental Study	China	Nanjing Youth Olympics, 2014	31	15 July 2014–30 September 2014	12	4
[43]	A panel study	China	Beijing Olympics, 2008	128	2 June 2008–30 October 2008	16	4
[44]	Time series study	China	Beijing Olympics and Paralympic games, 2008	10,948 (Number of cases for each sub-category of disease)	1 January 2007–31 December 2008	16 (Olympics) + 11 (Paralympics)	5
[45]	Time series study	China	Beijing Olympics, 2008	12,299,000 registered residents	20 May 2008–1 December 2008	16	5
[46]	A panel study	China	Beijing Olympics, 2008	111	2 June 2008–30 October 2008	16	4
[47]	A panel study	China	Beijing Olympics, 2008	36	11 June 2007–12 September 2008	16	4
[48]	Observational study	China	Asian Games, 2010	12.7 million permanent residents	1 January 2006–31 December 2011	15	5
[49]	A panel study	China	Beijing Olympics, 2008	125	20 July 2008–17 September 2008	16	4
[50]	A panel study	China	Beijing Olympics and Paralympic games, 2008	180	8 August 2008–17 September 2008	16 (Olympics) + 11 (Paralympics)	4
[51]	A panel study	China	Beijing Olympics, 2008	114	10 June 2008–31 October 2008	16	4
[52]	A panel study	China	Beijing Olympics, 2008	125	2 June 2008–30 October 2008	16	4
[53]	A panel study	China	Beijing Olympics, 2008	128	2 June 2008–30 October 2008	16	4
[54]	Observational study	China	Beijing Olympics, 2008	125	2 June 2008–30 October 2008	16	4
[55]	A panel study	China	Beijing Olympics, 2008	125	2 June 2008–30 October 2008	16	4
[56]	Observational study	China	Beijing Olympics and Paralympic games, 2008	1.818 million district population	1 January 2003–31 December 2008	16 (Olympics) + 11 (Paralympics)	6
[57]	Observational study	China	Beijing Olympics, 2008	N/A	20 July 2008–20 September 2008	16	4
[58]	A panel study	China	Beijing Olympics, 2008	36	11 June 2007–12 September 2008	16	5
[59]	Observational study	USA	Atlanta Olympics, 1996	10 million ED visits to 40 hospitals for the summers of 1993 through 2004	June 21–September 1 of years 1995–2004, including 1996	17	5
[60]	A panel study	China	Beijing Olympics, 2008	11	26 May 2008–14 November 2008	16	4

Abbreviations: N/A: Not Available, ED: emergency departments.

**Table 2:** Air pollution, mortality and hospitalization during mega-events: An overview of study designs and principal results.

Ref.	Air Pollution (µg/m^3^ or mg/m^3^) Before/During/After Mega-Event Mean, (SD); Median, [IQR]	Air Pollution Measurement Method	Outcomes Mean (Min–Max); Mean (95%; CI); (Mean ± SD); [Median, IQR]	Statistical Analyses	Results
[33]	**O_3_**, *Before*: 44.9 ± 17.4; *During*: 44.8 ± 10.5 **NO_2_**, *Before*: 114.6 ± 44.7; *During*: 120.6 ± 39.6,	Guangdong Environment Monitoring Center: NO_2_ and SO_2_: measured using chemiluminescence and fluorescence instruments	**MortalityTotal:** *Before-After period:* 121.7 ± 26.5; *During:* 102.9 ± 11.1, **Cardiovascular**: *Before-After period*: 49.2 ± 14; *During*: 38.9 ± 7.1, **Respiratory:** *Before-After period*: 21.3 ± 7.2; *During*: 17.3 ± 3.1	the short-term mortality effect for O_3_ and NO_2_ were assessed using **GAM** with **a quasi-Poisson** link.	↑ O_3_ (+10 µg/m^3^) → ↑ Total mortality (+0.60%; 95% CI: 0.47–0.74) ↑ NO_2_ (+10 µg/m^3^) → ↑ Total mortality (+1.89%; 95% CI: 1.49–2.29) ↑ O_3_ (IQR increase) → ↑ Cardio-respiratory mortality ↑ O_3_ (IQR increase) → ↑ Cardiovascular mortality (+4.42%; 95% CI: 3.16–5.69)
[44]	**PM_2.5_**, *Total period*: 78.0 ± 54.3 **PM_10_**, *Total period*: 136.0 ± 85.5	TEOM(R) RP1400A	**Hospitalization:** **Cardiovascular ERV** *Total period:* 15 ± 5	They applied a time-series analysis approach using **generalized additive Poisson regression models** to estimate the associations between air pollutants and cardiovascular ERV.	↑ Air pollution (non-Olympics period) → ↑ Cardiovascular ERV Olympics period (↓ air pollution) → No effect observed on cardiovascular ERV Effects of PM_2.5_ on cardiovascular ERV → more pronounced in females ↓ Air pollution during Olympics → ↓ Cardiovascular ERV Observed adverse effects of PM_2.5_ on cardiovascular ERV → An IQR increase (68 µg/m^3^) in PM_2.5_ associated with an overall relative risk of 1.022 (95% CI 0.990–1.057)
[45]	**PNC**, *before*: 2966 ± 1044; *During*: 2422 ± 994; *After*: 3641 ± 996 **PM_2.5_**, *Before*: 87.4 ± 41.0; *During*: 52.6 ± 27; After: 67.2 ± 47.7	A TDMPS system consisting of two Hauke-type DMAs and two CPCs (TSI model 3010 and TSI model 3025). Additionally, an APS measures number size distributions between 800 nm and 10 μm (aerodynamic diameter) accumulated over every 10 min.	**Mortality** **CVD**: Before: 41 ± 7; During: 42 ± 6; After: 54 ± 8	They investigated air pollution effects on daily CVD death counts for the whole study period using **quasi-Poisson regression models.**	↓ Air pollution (especially PNC) during Olympic & Paralympic Games → ↓ CVD mortality risk
[56]	**PM_10_**, Total period: 143.1 ± 87.2 **NO_2_**, *Total period*: 64.8 ± 24.2 **SO_2_**, *Total period*: 112.4 ± 316.9	The Beijing Municipal Environmental Protection Monitoring Center.	**Mortality** **Total deaths**, *Total period*: 22.8 ± 7.2 **Cardiovascular**, *Total period:* 10.4 ± 4.0 **Respiratory**, Total *period:* 2.2 ± 1.8	Because the daily number of deaths was small and typically followed a Poisson distribution, they used the **GAM** with penalized splines to examine the daily counts of mortality, air pollution and covariates.	↑ PM_10_ → Stronger effect on cardiovascular mortality than on respiratory mortality ↑ SO_2_ → Similar effect on cardiovascular and respiratory mortality ↑ NO_2_ → Stronger effect on respiratory mortality than on cardiovascular mortality
[59]	**PM_10_**; *Before*: 37.6 ± 14.2; *During*: 31.2 ± 10.4; *After*: 35.9 ±12.1 **O_3_**; *Before*: 76.3 ± 20.3); *During*: 53.6 ± 17.0; *After*: 68.9 ± 19.3 **NO_2_**; *Before*: 49.1 ± 15.9; *During*: 43.7 ± 8.17; *After*: 49.4 ± 15.6 **CO**; *Before*: 2.26 ± 1.38; *During*: 1.55 ± 0.43; *After*: 2.25 ± 1.40	The EPA Air Quality System and the Georgia Department of Natural Resources.	**Hospitalization ED visits:** **Respiratory diseases:** *During:* RR (95% CI) 0.901 (0.810–1.002) **Cardiovascular diseases:** *During:* RR (95% CI) 0.994 (0.878–1.125)	They used a Poisson GEE model with the daily number of ED visits as the outcome variable for each case group, and we included an offset of the log of the total non- accidental and non-case ED visits.	↑ O_3_ (per IQR increase) → ↑ Respiratory ED visits (especially upper respiratory infection & asthma) (+2% to +4%) ↑ PM, CO, NO_2_ → ↑ Cardiovascular ED visits
[30]	**PM_2.5_**; *Before:* 45 µg/m^3^, *During:* 34.7 µg/m^3^ The variation in **O_3_** concentration ranged from 23% to 6.6%	The National Urban Air Quality Real-time Distribution Platform of the China Environmental Monitoring General Station.	**Health risk:** **Premature deaths due to PM_2.5_ (numbers):** *Before:* 1939 *During:* 1780 **cardiovascular diseases deaths due to PM_2.5_ (numbers):** *Before:* 866, *During*: 795 **Premature deaths due to O_3_ (numbers)**: *Before*: 7134 *During*: 7332 **Cardiovascular diseases deaths due to O_3_ (numbers)**: *Before*: 3091, *During*: 3177	\	PM_2.5_ exposure during Asian Games → All-cause deaths: 1780 | Cardiovascular deaths: 795 PM_2.5_ (Asian Games) → ↓ 8.2% vs. same period 2022 O_3_ exposure during Asian Games → All-cause deaths: 7332 | Cardiovascular deaths: 3177 O_3_ (Asian Games) → ↑ 2.8% vs. same period 2022 ↑ of 1 µg/m^3^ in PM_2.5_ and O_3_ → ↑ in all-cause mortality of 16.1% and 21.9%, respectively.
[32]	**PM_2.5_**, *Before*: 87.4 ± 41.0; *During*: 52.6 ± 27.0; *After*: 67.2 ± 47.7 **PNC 100–300 nm (cm^−3^)**, *Before*: 4445 ± 1835; *During*: 3582 ± 1325; *After*: 5936 ± 2772 **PNC 300–1000 nm (cm^−3^)**, *Before*: 898 ± 529; *During*: 649 ± 421; *After*: 1106 ± 794	Monitoring was done using a TEOM(R) RP1400A air sampler. We obtained data on PM_10_ from the Beijing Municipal Environmental Monitoring Center’s network.	**Hospitalization:** **Respiratory diseases:***Before:* 11 ± 4, *During:* 10 ± 3, *After: 12 ± 3* **Pneumonia**: *Before*: 3 ± 2, *During*: 3 ± 2, *After*: 5 ± 2	Poisson regression models	Olympic & Paralympic Games → Improved air quality → ↓ Respiratory mortality Post-Olympics period (air pollution ↑) → Respiratory mortality remained ↓ Olympic period (strongest control measures) → Lowest pneumonia mortality risk Effects especially pronounced for PM_2.5_ and PNC (100–300 nm; 300–1000 nm) 7958 particles/cm^3^ in UFP→ ↑ respiratory mortality 16.3% PM_2_._5_ → No association with respiratory or pneumonia mortality
[34]	**PM_10_**, *Before-After:* 80.47 ± 24.12; *During:* 88.64 ± 43.23; **NO_2_**, *Before-After:* 64.27 ± 19.91; *During:* 66.44 ± 31.79; **SO_2_**, *Before-After:* 33.15 ± 16.67; *During:* 34.61 ± 27.23	The Guangdong Environmental Monitoring Center.	**Stroke Mortality:** **Total stroke:***Before-After*: 3.67 ± 2.07, *During:* 3 ± 1.43 **Ischemic stroke**: *Before-After*: 2 ± 1.49, *During*: 1.76± 1.17 **Hemorrhagic stroke:** *Before-After*: 1.67± 1.43, *During:* 1.24 ± 0.96	Student’s *t*-test was applied to examine the statistical differences, they also used interrupted time-series analysis based on Poisson regression.	2010 Guangzhou Asian Games → ↓ Daily air pollution levels → ↓ Stroke mortality count Air pollution control program → ↓ Stroke mortality (especially hemorrhagic stroke) Daily stroke mortality → 3.67 (before after period) → 3.0 (Asian Games) → ↓ 18.26%. Daily ischemic stroke mortality → 2.0 (before after) → 1.76 (Asian Games) → ↓ 12% Daily hemorrhagic stroke mortality → 1.67 (before after) → 1.24 (Asian Games) → ↓ 25.75% PM_2.5_ → Positively associated with stroke mortality counts SO_2_ → Positively associated with stroke mortality counts NO_2_ → Positively associated with stroke mortality counts PM_10_ (Asian Games) → Significant decrease in daily level
[35]	**PM_2.5_**, *Before*: 108.6 ± 21.2; *During*: 70.9 ± 10.4; *After*: 79.2 ± 33.2	FRM-approved instruments are deployed at the American Embassies in Beijing, which monitor and record data in real-time.	**Excess Mortality:** *Before*:4.2 ± 1.1; During: 2.3 ± 0.5; *After*: 2.7 ± 1.7	Derived from meta-analyses of different studies	↑ PM_2.5_ levels → ↑ Mortality ↓ PM_2.5_ (through mitigation actions) → ↓ Mortality
[39]	**PM_2.5_**, *Before*: 34 ± 4; *During*: 88 ± 16; *After*: 43 ± 6 **PM_10_**, *Before*: 87 ± 20; *During*: 176 ± 19; *After*: 43 ± 6	To measure PM, we utilized 4 fine particulate matter samplers running at a flow rate of 16.67 L per minute for 24 h sampling. Flows were checked pre- and postfilter exchange using a Mesa DryCal Defender 530 (Mesa Labs, Butler, NJ, USA).	**Hospitalization:** **Respiratory hospital admission due to PM_2.5_ (numbers):** *Before:* 34 out of 460, *During:* 88 out of 720, *After: 43 out of 567* **Respiratory hospital admission due to PM_10_ (numbers):** *Before*: 87 out of 460, *During*: 176 out of 720, *After*: 86 out of 567	\	Holi festival day → Respiratory hospital admissions ~2× (vs pre-Holi) Holi festival day → Respiratory hospital admissions ~1.3× (vs post-Holi) Holi festival day → Eye infection admissions 1.2× (vs pre-Holi) Holi festival day → Eye infection admissions 1.3× (vs post-Holi) ↑ PM_10_→ Greater health impact than PM_2.5_ (~3×) ↑ PM_10_ → ↑ Upper respiratory hospital admissions (more than PM_2.5_)
[48]	**PM10**, *Before-After:* 80.47 ± 24.12; *During:* 88.64 ± 43.23; **NO_2_**, *Before-After:* 64.27 ± 19.91; *During:* 66.44 ± 31.79; **SO_2_**, *Before-After:* 33.15 ± 16.67; *During:* 34.61 ± 27.23	The Environmental Monitoring Center	**Mortality:** **Non-accidental:** *Before-After*: 32 (7); *During*: 25 (5), **Cardiovascular:** *Before-After*: 11 (4); During: 8 (3), **Respiratory**: *Before-After:* 6 (3); *During:* 5 (2)	The association between air control measures and daily mortality was evaluated using a **time series Poisson** regression model.	Games year (2010) vs before 0 after period→ ↑ PM_10_ → No change in non-accidental and cardiovascular mortality → ↑ Respiratory mortality During 2010 Asian Games (mitigation period) → ↓ PM_10_ (88.64 → 80.61 µg/m^3^) ↓ PM_10_ during Games → ↓ All-cause mortality (32 → 25 per day) ↓ PM_10_ during Games → ↓ Cardiovascular mortality (11 → 8 per day) ↓ PM_10_ during Games → ↓ Respiratory mortality (6 → 5 per day)
[36]	**PM_2.5_ (µg/m^3^)**, *Before*: 132; *After*: 192 **PM_10_ (µg/m^3^)**, *Before*: 208; *After*: 245 **SO_2_ (µg/m^3^)**, *Before:* 7; *After: 15* **NO_2_ (µg/m^3^)**, *Before:* 87; *After:* 80	CPCB, 24 h ambient sampling	**Hospital admission:** Significant increase in cough post-Diwali in Kotla (6.7% → 28.9%); **respiratory admissions** increased in 45% of hospitals	Chi-square tests; comparisons of means (*p*-values reported), Hazard Quotient (HQ), Health Index (HI)	↑ PM_2.5_ and PM_10_ post-Diwali → ↑ Total hospital admissions (10 hospitals, 50%) ↑ PM_2.5_ and PM_10_ post-Diwali → ↑ Respiratory admissions (9 hospitals, 45%) ↑ PM_2.5_ and PM_10_ post-Diwali → ↑ Cardiac admissions (8 hospitals, 40%) ↑ PM_2.5_ and PM_10_ post-Diwali → ↑ Stroke admissions (4 hospitals; 20%) ↑ PM_2.5_ and PM_10_ post-Diwali → ↑ Respiratory symptoms (cough: 6.7% → 28.9% in Kotla) ↑ PM_2.5_ and PM_10_ post-Diwali → ↑ Eye problems (watering eyes: 21.3%)
[41]	**PM_10_**, *During*: 97.99 ± 36.22	Derived from API; daily API from 82 cities used to recover monthly PM_10_	**Mortality**: 10 µg/m^3^ decrease in PM_10_⇒ ↓ 8.4–9.6% all-cause mortality; ↓ 8.8% CVR deaths; Mortality rose slightly as PM_10_ rose again post-2008	Regression	Significantly lower mortality during → 10 µg/m^3^ decrease in PM_10_ → ↓ All-cause mortality (8.4–9.6%) → ↓ Cardiovascular mortality (8.8%) Before and after 2008 Olympics → ↑ PM_10_ → ↑ Mortality
[57]	**PM_2.5_** *non-Olympic period*: 93.9 ± 50.2 Olympics *period:* 64.7 ± 36.3 **PAH** *non-Olympic period*: mean = 11.1 ng/m^3^; Olympic *period:* BaPeq (ΣPAH17) mean = 4.33 ng/m^3^	the PM_2.5_ sample filters were extracted twice with dichloromethane (Optima; Fisher Scientific, Hampton, NH, USA) using pressurized liquid extraction (ASE 300; Dionex Corp., Sunnyvale, CA, USA). PAH measurement was analyzed by GC (Agilent 5890 GC; Agilent Technologies, Santa Clara, CA, USA) coupled to a quadrupole MS using electron impact ionization (70 eV; Agilent 5973 MSD)	**Cancer risk from Σ PAH17-BaPeq**: *Before*: 12.2–964 cases/million; During: 6.5–518 cases/million (depending on risk factor used)	Point-estimate approach using BaP-relative potency factor (RPF) and unit risk (UR) estimates from OEHHA and WHO	↓ PM_2.5_ → ↓ BaPeq concentration (−46%) → ↓ Cancer risk

Abbreviations: ↑: increase, ↓: decrease, →: indicate. O_3_:Ozone, NO_2_: nitrogen dioxide, PM_2.5_: Inhalable Particulate matter with a 2.5 microns or less in diameter, PM_10_: Inhalable Particulate matter with a diameter of 10 microns or less, PNC: Particle number concentration, CO: carbon monoxide, PAH: Polycyclic Aromatic Hydrocarbons, TEOM(R) RP1400A: air sampler (TEOM, model 1400A, Rupprecht and Patashnick; Thermo Electron, East Greenbush, NY, USA), TDMPS: Twin Differential Mobility Particle Sizer, APS: Aerodynamic Particle Sizer, TSI model 3321, GAM: the generalized additive model, BAM: Beta Attenuation Monitor, CVD: Cardiovascular diseases, API: Air Pollution Index; CPCB: Central Pollution Control Board, FRM: Federal Reference Method, ERV: Emergency Room Visits, GEE: generalized estimating Equations, OEHHA: Office of environmental health hazard assessment, WHO: World Health Organization.

**Table 3:** Air pollution and early biological responses during mega-events: An overview of study designs and principal results.

Ref.	Air Pollution (µg/m^3^ or mg/m^3^) Before/During/After Mega-Event Mean, (SD); Median, [IQR]	Air Pollution Measurement Method	Biomarkers Mean (95% CI) (Mean ± SD) [Median, IQR]	Biological Matrix Analytic Method	Statistical Analyses	Results
[31]	**PM_2__.5_**, *During:* 42.23 ± 21.56	PM_2.5_ by the personal monitored device (Ai 100, Huawei Technologies Co., Ltd., Shenzhen, China)	**Blood pressure:** **SBP**, *During*: 111.04, 9.26; **DBP**, *During*: 77, 6.53; **PP**, *During*: 34.04, 6.84; **MAP**, *During*: 88.35, 6.83	**Matrix:** Blood (blood pressure) **Method**: Electronic blood pressure monitor (Omron U10L, OMRON Healthcare Co., Ltd. China)	LME models to quantify the association between blood pressure and PM_2.5_ exposure.	↑ PM_2.5_(short-term exposure) → ↑ Blood pressure ↑ PM_2.5_ after WMG → ↑ SBP, ↑ DBP, ↑ PP
[37]	**PM_2.5,_** *Before*: 83.2; *During*: 32.7; *After*: 45.7 **PM_10_**, *Before*: 127.8; *During*: 55.9; *After*: 139.8	Particle mass monitor (Met one 531 AEROCET Particulate profiler, Met One Instruments, Inc. Grant Pass, OR, USA)	**GRO-α**, *Before:* 52.83 (44.33, 62.97), *During:* 41.45 (34.58, 49.68), *After:* 50.51 (42.81, 59.60) **IL-8**, *Before:* 53.11 (43.33, 65.09), *During*: 36.72 (28.54, 47.23), *After*: 42.39 (34.77, 51.69) **IP-10**, *Before:* 79.92 (72.72, 87.82), *During*: 80.38 (73.00, 88.51), *After:* 80.27 (72.63, 88.72) **MCP-1**, *Before:* 121 (109.90, 132.10), *During*: 130.28 (119.71, 140.84), *After:* 144.72 (133.60, 155.84) **RANTES**, *Before*: 27,202 (25437, 28966), *During*: 20,113 (18099, 22126), *After:* 29,424 (27710, 31137) **MCP-2**, *Before:* 43.39 (39.68, 47.10), *During*: 34.33 (30.82, 37.84), *After:* 46.33 (42.76, 49.90) **Eotaxin-1**, *Before:* 138.61 (125.87, 152.64), *During*: 132.27 (119.80, 146.04), *After:* 153.87 (140.21, 168.86) **TARC**, *Before:* 172.94 (155.41, 192.43), *During*: 111.89 (95.94,130.50), *After:* 180.80 (165.08, 198.01)	**Matrix:** Blood (serum) **Method**: Q-PlexTM Human Chemokine ELISA-based chemiluminescent assay (Quansys Biosciences, Logan, UT, USA)	Linear mixed effects regression models were used to compare serum concentrations of chemokines across the three time periods.	↓ Air pollution during Olympic period → ↓ RANTES, ↓ MCP-2, ↓ TARC ↑ Air pollution after Games → ↑ RANTES, ↑ MCP-2, ↑ TARC ↑ Air pollution → Positive association with IL-8, Eotaxin-1, GRO-α (not always statistically significant)
[40]	**PM_2.5_**, *Before*: 83.2; *During*: 32.7; *After*: 45.7 **PM_10_**, *Before*: 127.8; *During*: 55.9; *After*: 139.8	Particle mass monitor (Met one 531 AEROCET Particulate profiler, Met One Instruments, Inc. Grant Pass, OR, USA)	**GPx**, *Before:* 900.81 (880.04, 921.58), *During*: 798.67 (776.93, 820.42), *After:* 857.26 (835.13, 879.39) **GR**, *Before:* 6.52 (6.30, 6.73), *During:* 6.45 (6.19, 6.71), *After:* 6.37 (6.14, 6.59) **GST**, *Before:* 15.63 (15.13, 16.13), *During:* 15.27 (14.74, 15.81), *After:* 15.54 (15.00, 16.07) **TAS**, *Before***:** 1.01 (0.97, 1.06), *During:* 0.95 (0.91, 0.99), *After*: 0.91 (0.87, 0.94)	**Matrix**: Blood (serum) **Method:** GST,GPx, GR with automated enzyme kinetic methodology adapted to the Cobas Fara II automated, centrifugal analyzer; TAS with the Randox TAS kit adapted to the Cobas Fara II autoanalyzer	Univariate analyses were performed to describe participant demographic information. Continuous variables, means (SD) and their 95% confidence intervals were reported for GPx, GR, GST, and TAS.	↓ Air pollution during Games → ↓ GPx activity ↑ Air pollution after Olympics → ↑ GPx activity post-Olympics GPx increase → Smaller in males, older participants, smokers (vs females, younger, nonsmokers) ↓ Air pollution during Games → ↓ TAS (all participants) ↑ Air pollution after Olympics → ↑ TAS in females, younger participants, nonsmokers ↑ Air pollution after Olympics → TAS continued ↓ in males, older participants, smokers Changes in air pollution → No significant response in GR and GST
[42]	**PM_2.5,_** *Before*: 53.6 ± 24.5, *During*: 37.3 ± 12.5, *After*: 38.1 ± 18.5 **SO_2_**, *Before:* 15.2 ± 4.1, *During*: 12.9 ± 3.7, *After:* 18.1 ± 10.5 **NO_2_** *Before:* 22.0 ± 6.6, *During*: 24.1 ± 5.9, *After* 27.0 ±8.1 **O_3_**, *Before:* 40.6 ± 14.9, *During*: 19.2 ± 15.1 *After:* 64.4± 18.3 **CO**, *Before:* 1.1 ± 0.2; *During*: 1.1 ± 0.2; *After:* 1.1 ± 0.2	Hourly air quality data, from a fixed-site monitoring station about 5 km away from the community health center.	**sCD40L**, *Before*: 4.23 ± 1.89, *During:* 3.38 ± 2.35, *After*: 3.60 ± 2.67 **MCP-1**, *Before:* 0.49 ± 0.25, *During:* 0.48 ± 0.25, *After*: 0.50 ± 0.22 **TNF-α**, *Before:* 10.27 ± 10.66), *During:* 10.76 ± 14.17, *After*: 9.84 ± 11.91 **CRP**, *Before:* 1.43 ± 7.17, *During:* 1.28 ± 6.56, After: 1.34 ± 4.19 **ICAM-1**, *Before:* 79.28 ± 40.94, *During:* 81.65 ± 34.70, *After:* 80.11 ± 37.38 **P-selectin**, *Before:* 50.44 ± 28.55, *During:* 46.08 ± 18.32, *After*: 45.97 ± 23.98 **VCAM-1**, *Before:* 346.13 ± 118.69, *During:* 322.37 ± 113.40, *After:* 350.23 ± 108.97 **IL-1β**, *Before:* 0.07 ± 0.12; *During:* 0.05 ± 0.08, *After:* 0.06 ± 0.07	**Matrix**: Peripheral blood **Method:** We measured the biomarkers using enzyme-linked immunosorbent assays or the MilliporeMILLIPLEX MAP human cytokine chemokine kit (Millipore Corporation, Billerica, Massachusetts).	A linear mixed-effect model to evaluate the associations between air quality and biomarkers was used. All statistical tests were 2-sided, and a P value less than 0.05 was considered statistically significant.	Before, during, after-Olympic periods → Significant changes in sCD40L, IL-1β, CRP, VCAM-1 ↑ PM_2.5_ and ↑ O_3_ → ↑ sCD40L, ↑ ICAM-1, ↑ VCAM-1, ↑ P-selectin, ↑ IL-1β ↓ PM_2.5_ and ↓ O_3_ → ↓ Inflammatory biomarkers PM_2.5_ and O_3_ → Significant association with serum levels of sCD40L, ICAM-1, P-selectin, VCAM-1, IL-1β
[43]	**PM_2.5_**, *Before:* 98.9 ± 14.7, *During*: 71.9 ± 15.1, *After*: 85.3 ± 15.3 **CO**, *Before*: 1.23 ± 0.13, *During:* 0.64 ± 0.14, *After:* 0.81 ± 0.14 **NO_2_**, *Before:* 25.60 (3.66), *During*: 14.61 ± 3.76, *After*: 41.39 ± 3.81 **O_3_**, *Before:* 31.84 ± 3.75, *During*: 39.60 ± 3.85, *After*: 15.12 ± 3.91 **SO_2_**, *Before:* 7.45 ± 1.17; *During*: 2.97 ± 1.33, *After*: 6.81 ± 1.22	All the air samplers and monitors were collocated at a secured spot on the Peking University First Hospital campus that served as the clinical base.	**EBC nitrite (μmol/L)**, *Before:* 7.33, *During*: 4.71, After: 4.71 **eNO (p.p.m.)**, *Before:* 11.75, *During*: 5.80, *After:* 12.47 **vWF (% of normal)**, *Before:* 102.6, During: 90.2, *After:* 83.8 **sCD62p (ng/mL)**, *Before:* 6.50, *During:* 5.01, *After:* 5.34 **sCD40L (ng/mL)**, *Before:* 1.8, *During:* 1.74, *After:* 1.91 **8-OHdG (mg/mol creatinine)**, *Before:* 3.32, *During*: 1.62, *After*: 3.05	**Matrix**: Blood **Method**: HPLCwith an UV detector for EBC nitrite, HPLC with an ECD for 8-OHdG, a chemiluminescense analyzer for eNO, and ELISA for vWF, sCD62P, and sCD40L.	Statistical analyses were conducted using R version 2.14.2 (Platform: i386-pc-mingw32/i386 (32-bit).	Olympic period (vs before) → ↓ EBC nitrite, ↓ eNO, ↓ sCD62P, ↓ sCD40L, ↓ 8-OHdG Post-Olympics (pollution ↑) → ↑ EBC nitrite, ↑ eNO, ↑ sCD62P, ↑ sCD40L, ↑ 8-OHdG Olympic period (vs before) → ↓ vWF → continued ↓ after Olympics
[46]	**PM_2.5_**_,_*Before:* 98.9 ± 14.7, *During*: 71.9 ± 15.1, *After*: 85.3 ± 15.3 **CO**, *Before*: 1.23 ± 0.13, *During:* 0.64 ± 0.14, *After:* 0.81 ± 0.14 **NO_2_**, *Before:* 25.60 (3.66), *During*: 14.61 ± 3.76, *After*: 41.39 ± 3.81 **O_3_**, *Before:* 31.84 ± 3.75, *During*: 39.60 ± 3.85, *After*: 15.12 ± 3.91 **SO_2_**, *Before:* 7.45 ± 1.17; *During*: 2.97 ± 1.33, *After*: 6.81 ± 1.22	PM_2.5_ were collected on a 37 mm Teflon filter using a Quad Channel Ambient Particulate Sampler equipped with an impactor that has an aerodynamic cut-off of 2.5 μm (TH-16A, Tianhong Inc., Wuhan,China) at a flow rate of 16.7 L/min.	*Before: Creatinine-standardized* **1&2-Amino-naphthalene**, **pg/μg creatinine** 0.48 (0.36, 0.65) **1-Amino-pyrene**, **pg/μg creatinine** 0.16 (0.11, 0.22) **1-Hydroxy-pyrene**, **pg/μg creatinine** 0.53 (0.44, 0.63) *During: Creatinine-standardized* **1&2-Amino-naphthalene**, **pg/μg creatinine** 0.47 (0.36, 0.63) **1-Amino-pyrene**, **pg/μg creatinine** 0.19 (0.14, 0.27) **1-Hydroxy-pyrene**, **pg/μg creatinine** 0.50 (0.42, 0.60) *After:Creatinine-standardized* **1&2-Amino-naphthalene**, **pg/μg creatinine** 0.61 (0.38, 0.97) **1-Amino-pyrene**, **pg/μg creatinine** 0.25 (0.15, 0.42) **1-Hydroxy-pyrene**, **pg/μg creatinine** 0.46 (0.35, 0.60)	**Matrix:** Urine **Method**: HPLC-fluorescence system for the detection of 1&2-amino- naphthalene (as one single peak without resolving 1-AN and 2- AN), 1-amino-pyrene, and 1-hydroxy-pyrene.	Linear mixed-effects models were used to estimate the changes in urinary amino-PAH levels across the three sampling periods (pre-, during-, and post-Olympic period).	Before Olympics to During-Olympic → ↓ 1&2-amino-naphthalene (−23%) → ↓ 1-hydroxy-pyrene (−16%) → No change in 1-amino-pyrene (+2%) During- to After Olympic → ↑ 1&2-amino-naphthalene (+26%) → ↑ 1-amino-pyrene (+37%) → ↑ 1-hydroxy-pyrene (+3%) 1&2-amino-naphthalene and 1-hydroxy-pyrene → Associated with traffic-related pollutants (similar lag pattern) 1-amino-pyrene → Associated with diesel combustion (PN, elemental carbon) → Not affected by season Exposure lag 24–72 h → Strongest associations 1&2-amino-naphthalene and 1-hydroxy-pyrene → Biomarkers of exposure to vehicle-emitted pollutants
[47]	**PM_2.5_**, *Before:* 161.5 ± 94.8, *During*: 47.8 ± 26.03, **CO**, *Before*: 1.69 ± 1.24, *During:* 0.87 ± 0.28, **NO_2_**, *Before:* 31.142 ± 13.51, *During*: 25.746 ± 2.97, **O_3_**, *Before:* 34.64 ± 28.517, *During*: 40.72 ± 29.0, **SO_2_**, *Before:* 27.89 ± 20.19; *During*: 13.9 ± 3.392, **BC**: *Before*: 5.20 ± 3.10; *During*: 1.8 ± 0.51,	Air pollutant concentrations were measured continuously at the air pollution monitoring station located southwest of the elementary school.	**8-oxodG ng/mg creatinine**: *Geometric mean*: *Before*: 2.30, After: 1.43 **Malondialdehyde mmol/mol creatinine***: Geometric mean*: Before: 0.108, After: 0.83	**Matrix:** Urine **Method**: 8-oxodG was measured using HPLC equipped with an electrochemical detector (Model 2465, Waters, Milford, MA, USA). MDA was determined by HPLC-UV (Waters 2998, Milford, MA, USA). Urinary creatinine levels were analyzed by a commercial kit (Jiancheng Bio-engineering Institute, Nanjing, China).	Repeated measures one-way ANOVA; GEE with log-transformed biomarkers	↑ BC, PM_2.5_, SO_2_, NO_2_, CO → ↑ MDA → ↑ 8-oxodG
[49]	**PM_2.5_**, *Before:* 98.9 ± 14.7, *During*: 71.9 ± 15.1, *After*: 85.3 ± 15.3 **CO**, *Before*: 1.23 ± 0.13, *During:* 0.64 ± 0.14, *After:* 0.81 ± 0.14 **NO_2_**, *Before:* 25.60 ± 3.66, *During*: 14.61 ± 3.76, *After*: 41.39 ± 3.81 **O_3_**, *Before:* 31.84 ± 3.75, *During*: 39.60 ± 3.85, *After*: 15.12 ± 3.91 **SO_2_**, *Before:* 7.45 ± 1.17; *During*: 2.97 ± 1.33, *After*: 6.81 ± 1.22	-PM_2.5_ collection using Quad-Channel ambient particulate sampler (TH-16A) with Teflon and quartz filters Sulfate analysis by ion chromatography on quartz filters -EC and OC measured by NIOSH Method 5040 on heat-treated quartz fiber filters -SO_2_, NO_2_, CO, O_3_ using calibrated monitors following manufacturer protocols.	**Pulmonary inflammation and oxidative stress** **Autonomic**, B*efore*: 0.001, 0.012, After*:* 20.016, 0.007. *p* value = 0.0568 **Hemostasis**, *Before*: 0.004, 0.026, *After:* 20.069, 20.026. *p* value < 0.0001 **Pulmonary**, *Before:* 20.019, 0.004, After*:* 20.035, 0.001. *p* value = 0.3877 **Systemic**, *Before*: 20.009, 0.004, *After:* 20.027, 0.003. *p* value = 0.1741	**Matrices:** Blood and Urine **Methods:** Autonomic function: 12-lead 3-channel MGY-S2 ECG system with ECG Lab 3.0 software. Blood pressure measured by manual sphygmomanometer after 5 min rest sCD62P and sCD40L measured by ELISA VWF measured by commercial ELISA kit Plasma fibrinogen measured by automated ACL9000 analyzer Red and white blood cell counts Urinary 8-OHdG measured by HPLC with electrochemical detection.	Exploratory univariate and bivariate analyses for outliers and confounders.	↑ Air pollution → ↑ Pulmonary inflammation and ↑ Oxidative stress biomarkers (within 24 h) ↑ Air pollution → ↑ Hemostasis biomarkers (after 2–3 days)
[50]	**PM_1_**, *Before:* 24 (20.0), *During:* 11 (13.3), *After:* 14 (16.90) **PM_2.5_**, *Before*: 83 (92.7), *During*: 33 (48.7), *After*: 46 (57.2) **PM_7_**, *Before*: 116 (117.0), *During*: 49 (59.1), *After*: 106 (96.7) **PM_10_**, *Before:* 128 (122.0), *During*: 56(60.4), *After*: 140 (120.7) **TSP**, *Before*: 141 (126.9), *During*: 63(61.7), *After*: 187 (157.9)	Particle mass monitor (Met One^®^ 531 AEROCET Particulate Profiler, Met One Instruments, Inc. Grant Pass, OR, USA) was used to measure the concentration of particulate matter in the study area, which was located in the center of the community.	**The breath rates at the pre-Olympic level (mean values: 19.3/min for females**, **19.0/min for males)** Diastolic pressure among males increased during the games from the pre-Olympic level by 4.04 mmHg (95CI: 1.90, 6.18). **The breath rate during the Olympics means values: 18.6/min for females**, **18.5/min for males** The pulses pressure decreased among males during the games from the pre-Olympic level by 2.35 mmHg (95CI: −4.58, −0.12). **The breath rate after the Olympics (mean values: 19.5/min for females**, **19.6/min for males).** The increased diastolic pressure maintained after the games (mean value: 86.21 mmHg). **The percentage of participants who had peak** **expiratory flow** greater than 400 L/min increased from 30% in the pre-Olympic period to 49.4% in the during-Olympic period and decreased to 33.4% in the post-Olympic period.	Peak expiratory flow was measured by a pre-calibrated peak flow meter.	Univariate analyses were conducted to characterize air pollution levels, participant demographic information and physical measurements.	↓ PM_1_, PM_2.5_, PM_7_, PM_10_, TSP during Olympics → ↓ Breath rate After Olympics (PM ↑ again) → ↑ Breath rate ↓ PM during Olympics → ↓ % with fast breath rate (>20/min) → ↑ post-Olympics During and After Olympics → ↑ Diastolic pressure (males) During and After Olympics → ↓ Pulse pressure (males) Air pollution variation → No clear pattern for overall blood pressure
[52]	**PM_2.5_**, *Before:* 98.9 ± 14.7, *During*: 71.9 ± 15.1, *After*: 85.3 ± 15.3 **CO**, *Before*: 1.23 ± 0.13, *During:* 0.64 ± 0.14, *After:* 0.81 ± 0.14 **NO_2_**, *Before:* 25.60 (3.66), *During*: 14.61 ± 3.76, *After*: 41.39 ± 3.81 **O_3_**, *Before:* 31.84 ± 3.75, *During*: 39.60 ± 3.85, *After*: 15.12 ± 3.91 **SO_2_**, *Before:* 7.45 ± 1.17; *During*: 2.97 ± 1.33, *After*: 6.81 ± 1.22	TDMPS	**HR (bpm)**, *Before*: 66.5 ± 1.0, *During*: 65.4 ± 1.0, *After*: 66.1 ± 1.0 **Urinary 8-OHdG (mg/mol creatinine)**, Before: 2.16 ± 1.81, During: 0.90 ± 1.95, After: 3.74 ± 1.46 **FeNO (ppb)**, *Before:* 11.53 ± 1.07, During: 4.58 ± 1.10, After: 10.52 ± 1.13 **EBC Nitrite (μM)**, *Before:* 6.30 ± 1.06, During: 1.28 (6.56), After: 1.34 (4.19) **EBC Nitrate (μM)**, *Before:* 2.84 ± 1.08, During: 2.23 ± 1.13, After: 5.82 ± 1.22 **8-Isoprostane (% ≥ 1.56 pg/mL)**, *Before:* 68, During: 44, After: 74 **sCD62P (ng/mL)**, *Before:* 6.49 ± 1.02, During: 5.03 ± 1.02, After: 5.34 ± 1.02 **sCD40L**, **ng/mL**, *Before*: 1.89 ± 1.02, During: 1.77 ± 1.02, After:1.90 ± 1.02 **vWF (%)**, Before: 102.1 ± 2.5, During: 90.0 ± 2.55, After: 83.8 ± 2.5 **Fibrinogen (g/L)**, *Before:* 2.46 ± 0.03, During: 2.41 ± 0.03, After: 2.83 ± 0.03	**Matrices:** Blood, urine, EBC **Methods**: EBC collection: Jaeger EcoScreen EBC collector 8-isoprostane in EBC: ELISA EBC nitrite and nitrate: HPLC with UV detector FeNO: Offline sampling method following ATS/ERS guidelines HR, HRV: 12-lead 3-channel MGY-S2 ECG Analysis System (ECG Lab 3.0) vWF in plasma: ELISA kit sCD62P and sCD40L in plasma: ELISA kits Urinary 8-Hydroxy-2′-deoxyguanosine: HPLC with ECD system	Descriptive statistics for each air pollutant and biomarker Calculation of means, standard deviations, quartiles, minimums, and maximums for each visit/period (pre-, during-, post-Olympics). Calculation of proportion of measurements above the minimum detectable level Values below detection limit replaced with half of the detectable limit for calculations.	During Olympics (pollution ↓) → ↓ Inflammatory, ↓ Oxidative stress, ↓ Hemostasis markers → Improved cardiovascular physiology After Olympics (pollution ↑) → ↑ Pulmonary inflammation markers (FeNO, EBC nitrite, nitrate, nitrite nitrate, pH, 8-isoprostane) After Olympics (pollution ↑) → ↑ Systemic inflammation/oxidative stress markers (fibrinogen, vWF, urinary 8-OHdG) After Olympics (pollution ↑) → ↑ Hemostasis/platelet activation markers (sCD40L, sCD62P, vWF) After Olympics (pollution ↑) → ↑ Cardiovascular measures (HR, SBP)
[53]	**PM_2.5_**, *Before:* 100.9 (38.8); *During:* 69.4 (42.9); *After:* 84.2 (67.2) **SO_2_**, **ppb**, *Before:* 7.6 (4.5); *During:* 3.1 (1.6); *After*:6.6 (3.6) **EC**, *Before*: 2.2 (0.7); *During:* 1.4 (0.6); *After:* 3.3 (1.6) **CO ppm**, *Before:* 1.25 (0.41); *During:* 0.63 (0.22); *After:* 0.82 (0.59) **NO_2_ ppb**, *Before:* 26.0 (5.1); *During:* 13.9 (4.6); *After:* 41.4 (17.2) **O_3_ ppb**, *Before:* 31.8 (16.4); *During:* 39.5 (16.0); *After:* 15.3 (6.5)	**PM_2.5_** concentration: Collected on Teflon filters Measured gravimetrically Sulfate: Analyzed from Teflon filters by ion chromatography **EC and OC:** Collected on heat-treated quartz fiber filters Measured using NIOSH Method 5040 in a commercial laboratory **SO_2_**, **NO_2_**, **CO**, **O_3_:** Measured EC9800 series ambient gas analyzers (Ecotech Ltd., Melbourne, Australia.)	**Oxidative stress:** **EBC MDA (nM)**, *Before:* 21.0 ± 1.5, During: 16.0 ± 1.9, After: 22.3 ± 2.4 **Urinary MDA (nM)**, **Before**: 435.3 ± 1.1, During: 311.1 ± 1.1; After: 483.0 ± 12.8	**Matrices:** EBC and urine **Methods:** samples EBC were collected using a Jaeger EcoScreen condensing device (Jaeger, Wurzburg, Germany). The method for analyzing MDA in EBC and urine samples was modified from a published method that used an HPLC system with fluorescent detection.	Linear mixed-effects models were described in a related, recently published paper to examine differences in MDA levels between periods (pre-, during-, and post-Olympics) as well as, separately, associations between pollutants and MDA.	↓ Air pollution (during Olympic controls) → ↓ EBC MDA ↑ Air pollution (after controls ended) → ↑ EBC MDA ↑ Air pollution (same day or previous days) → ↑ EBC MDA ↑ Air pollution (same day or previous days) → ↑ Urinary MDA
[54]	**PM_2.5_**, *Before:* 98.9 ± 14.7, *During*: 71.9 ± 15.1, *After*: 85.3 ± 15.3 **CO**, *Before*: 1.23 ± 0.13, *During:* 0.64 ± 0.14, *After:* 0.81 ± 0.14 **NO_2_**, *Before:* 25.60 (3.66), *During*: 14.61 ± 3.76, *After*: 41.39 ± 3.81 **O_3_**, *Before:* 31.84 ± 3.75, *During*: 39.60 ± 3.85, *After*: 15.12 ± 3.91 **SO_2_**, *Before:* 7.45 ± 1.17; *During*: 2.97 ± 1.33, *After*: 6.81 ± 1.22	**PM_2.5_** concentration: Collected on Teflon filters Measured gravimetrically Sulfate: Analyzed from Teflon filters by ion chromatography **EC and OC:** Collected on heat-treated quartz fiber filters Measured using NIOSH Method 5040 in a commercial laboratory **SO_2_**, **NO_2_**, **CO**, **O_3_:** Measured with calibrated continuous monitors (Ecotech Ltd.) **Sampling site:** Rooftop of a 7-story building at the hospital campus	**FENO**, **ppb**, *Before*: 11.76 ± 1.04, During: 5.80 ± 1.04, After: 12.51 ± 1.04, **EBC nitrite**, **mM**, *Before:* 7.33 ± 1.03, During: 4.71± 1.03, After: 4.69 ± 1.04 **EBC nitrate**, **mM**, *Before*: 2.79 ± 1.04, During:2.61 ± 1.0, After: 4.23 ± 1.05 **EBC nitrite 1 nitrate mM**, *Before*: 10.48 ± 1.03,*During*: 7.68 ± 1.03, *After*: 10.37 ± 1.03, **EBC pH**, *Before*: 7.43 ± 0.03, *During*: 7.46 ± 0.03, *After*: 7.61 ± 0.03 **EBC 8-isoprostane**, **% >DL 1.56 pg/mL**, *Before*: 68, *During*:44, *After*: 74 **Urinary 8-OHdG**, **mg/mol**, *Before*: 3.70 ± 1.12; *During:* 2.22 ± 1.12; *After:* 3.34 ± 1.12	**Matrices:** FENO, EBC and urine **Method**: sample FeNO: Measured by offline sampling following ATS/ERS guidelines. EBC collection: Using Jaeger EcoScreen collector. EBC nitrite and nitrate: Analyzed by HPLC-UV at 214 nm. EBC 8-isoprostane: Measured using ELISA assay (detection limit 1.56 pg/mL). Urinary 8-OHdG: Measured by HPLC with electrochemical detection.	Used time-series regression models to estimate period-specific means and differences in air pollutants and meteorologic variables. Estimated biomarker changes across periods (pre-, during-, post-Olympic) using linear mixed models for all biomarkers except 8-isoprostane.	↓ Air pollution (Olympic control) → ↓ FeNO → ↓ Pulmonary inflammation ↓ Air pollution (Olympic control) → ↓ Oxidative stress biomarkers ↑ Air pollution (0–48 h before) → ↑ FeNO → ↓ EBC pH → ↑ EBC nitrite → ↑ EBC nitrate ↑ Air pollution (48–96 h before) → ↑ EBC 8-isoprostane → ↑ DNA oxidative damage
[55]	**PM_2.5_**, *Before:* 100.9 (38.8); *During:* 69.4 (42.9); *After:* 84.2 (67.2) **SO_2_**, **ppb**, *Before:* 7.6 (4.5); *During:* 3.1 (1.6); *After*: 6.6 (3.6) **EC**, *Before*: 2.2 (0.7); *During:* 1.4 (0.6); *After:* 3.3 (1.6) **CO ppm**, *Before:* 1.25 (0.41); *During:* 0.63 (0.22); *After:* 0.82 (0.59) **NO_2_ ppb**, *Before:* 26.0 (5.1); *During:* 13.9 (4.6); *After:* 41.4 (17.2) **O_3_ ppb**, *Before:* 31.8 (16.4); *During:* 39.5 (16.0); *After:* 15.3 (6.5)	**PM_2.5_** concentration: Collected on Teflon filters Measured gravimetrically Sulfate: Analyzed from Teflon filters by ion chromatography **EC and OC:** Collected on heat-treated quartz fiber filters Measured using NIOSH Method 5040 in a commercial laboratory **SO_2_**, **NO_2_**, **CO**, **O_3_:** Measured with calibrated continuous monitors (Ecotech Ltd.)	**HR (bpm)**, Before: 66.5 (65.0 to 68.1), During: 65.4 (63.8 to 67.0), After: 66.1 (64.2 to 68.1) **sCD62P (ng/mL)**, Before: 6.29 (5.97 to 6.63), During: 4.16 (3.86 to 4.48), After: 5.36 (5.10 to 6.05) **sCD40L**, **ng/mL**, Before: 1.86 (1.79 to 1.94), During: 1.76 (1.66 to 1.86), After: 1.92 (1.77 to 2.07) **vWF (%)**, Before: 106.4 (98.5 to 114.40), During: 92.6 (82.6 to 102.5), After: 79.5 (66.9 to 92.1) **Fibrinogen (g/L)**, Before: 250 (242 to 258),During: 250 (242 to 259), After: 261 (249 to 273), **Blood pressure**, **mm hg Systolic**, Before: 102.5 (99.9 to 105.2), During: 100.9 (97.4 to 104.4), After: 110.5 (105.9 to 115.0) **Blood pressure**, **mm hg Diastolic**, Before: 60.2 (57.9 to 62.6); During:60.1 (57.0 to 63.1), After:60.1 (56.2 to 64.0)	**Matrix**: blood **Method:** Electrocardiographic measurements (HR) performed in the supine position. Blood pressure (systolic and diastolic) measured manually with a sphygmomanometer. Plasma sCD62P and sCD40L measured by commercial ELISA (Rapidbio). Plasma fibrinogen analyzed within 4 h using an automated ACL9000 analyzer. Plasma von Willebrand factor measured by commercial ELISA kit (Hushang Biotech). CRP measured in EDTA plasma via immunonephelometric assay with automated nephelometer.	Calculated descriptive statistics for each pollutant by period. Log-transformed sCD62P and sCD40L due to right-skewed distributions. Used Akaike Information Criterion (AIC) to select the best model for within-participant error correlation.	During Olympic Games → ↓ Particulate & gaseous pollutants (−13% to −60%) → ↓ Fibrinogen, ↓ von Willebrand factor, ↓ Heart rate, ↓ sCD62P, ↓ sCD40L After Olympic period → ↑ Pollutant concentrations (back to pre- Olympic levels) → ↑ Fibrinogen, ↑ von Willebrand factor, ↑ Heart rate, ↑ sCD62P, ↑ sCD40L After Olympic vs. During-Olympic → sCD62P and Systolic blood pressure significantly worse
[58]	**PM_2.5_ (μg/m^3^)**, *Before:* 183.4 ± 70.1 (μg/m^3^); During: 46.4 ± 26.1 **BC (μg/m^3^)** Before: 3.57 ± 1.88; During: 1.80 ± 0.48 **CO (ppm)** Before: 1.25 ± 0.46; During: 0.88 ± 0.29 **SO_2_ (ppb) Before**: 1.15 ± 0.63; During: 5.18 ± 3.55 **NO_2_ (ppb)** Before: 26.55 ± 6.96; During: 25.85 ± 2.89	Hourly averaged concentrations of PM_2.5_ (tapered element oscillating microbalance, RP1400a; ThermoScientific), BC (multi-angle absorption photometer, model 5012; ThermoScientific), NOx, SO_2_, and CO (models 9841A, 9850A, 9830A; ECOTECH Pty Ltd., Knoxfield, Australia).	**eNO (ppb)**, Before: 13.4 ± 7.5; During: 9.0 ± 5.8	**Matrix:** EBC **Method:** Collection device made of Teflon, equipped with a flow meter and pressure indicator. eNO concentrations measured within 4 h in a 4 L air-sampling bag. Analysis performed with a chemiluminescence NO–NO_2_–NOx analyzer (ThermoScientific model 42i Rockford, IL, USA).	*t*-Tests were used to derive *p*-values for differences in mean air pollutants and eNO before and during the Olympic Games air quality intervention. GEE To check the Assessed validity of correlations between repeated measurements using the quasi-likelihood under the QIC.	↑ BC → ↑ eNO (immediate effect and lag up to 10 h) ↑ BC and ↑ PM_2_._5_ → Smaller eNO response in asthmatic children ↑ BC → ↑ Respiratory inflammation (stronger effect than other pollutants)
[60]	**PM _2.5_**, *Before*:95.4 ± 58.6; *During*: 39.5 ± 25.2; *After*: 64.0 ± 80.3 **CO**, *before*:3.6 ± 1.4; *During*: 2.8 ± 1.0; *After*: 2.7 ± 0.7 **NO**, *before*: 36.4 ± 12.3; *During*: 30.3 ± 12.2; *After*:37.1 ± 17.0 **NO_2_**, *before*:176.1 ± 84.8; *During*: 156.0 ± 77.2; *After*: 268.0 ± 55.5	PM_2.5_ were measured by a portable aerosol spectrometer (model 1.109; Grimm Technologies Inc., Douglasville, GA, USA); CO was measured with a model T15n enhanced CO measurer (Langan Products Inc., San Francisco, CA, USA), and NO_2_ and NO were measured using a passive sampler (Ogawa Air Inc., Osaka, Japan).	**5 min SDNN (msec)** Before: 1320; During: 1366; After: 1309, **5 min LF power (msec2)** Before: 1298; During: 1360; After: 1287 **5 min HF power (msec2)** Before: 1298; During: 1360; After: 1287	**Matrix:** Blood **Method**: A three-channel Holter recorder (model MGY-H7; Holter System, version 12.net; DM Software Inc. Inc., Stateline, NV, USA) was used for ambulatory ECG monitoring.	All statistical analyses were performed using SAS software for Windows (version 9.1; SAS Institute Inc., Cary, NC, USA); HRV indices were log10-transformed to improve normality and stabilize the variance.	↑ PM exposure (high traffic) → Altered heart rate variability (HRV) in taxi drivers
[51]	**PM_2.5_ values in 6 stages at pre-Olympics**, **during-Olympics and post-Olympics:** (101.84 ± 52.64); (101.86 ± 25.76); (87.15 ± 51.13); (71.69 ± 40.52); (87.47 ± 79.36); (76.11 ± 60.58)	Air quality monitoring station by College of Environmental Sciences and Engineering, Perking, University	**vWF 6 different stages at pre-Olympics**, **during-Olympics and post-Olympics:** (103.93 ± 11.13); (100.16 ± 9.89); (91.70 ± 13.01); (85.02 ± 18.16); (88.46 ± 13.37); (76.35 ± 13.76)	**Matrix:** Blood (plasma) **Method**: ELISA kit (Hushang BioTech, Shanghai, China)	Student *t* test to evaluate plasma vWF levels in different stages. Correlation analysis between PM_2.5_ and Plasma	↑ PM_2.5_ → ↑ plasma vWF before & after measurements
[38]	**PM_2.5_ µg/m^3^**, *Before*: 83 ± 92.7; *During*: 33 ± 48.7; After:46 ± 57.2 **PM_10_ µg/m^3^**, *Before*: 128 ± 122.0; During: 56 ± 60.4; After: 140 ± 120.7	Met Ones 531 AEROCET Particulate Profiler (Met One Instruments, Inc. Grant Pass, OR, USA)	**Metabolomics changes associated with oxidative stress**, **inflammation**, **antioxidant activity: Lipids (PUFAs**, **LCFAs**, **lysolipids**, **eicosanoids)**, **dipeptides**, **amino acids (taurine**, **SAH)**, **nucleotides (xanthine**, **hypoxanthine)** **Before:** Elevated metabolite levels (e.g., EPA, 12-HETE, xanthine) before the Olympics; **During**: Decreased levels of identified biomarkers during the Olympics across all four significant metabolite modules; **After**: Increased levels again after the Olympics, consistent with pollution returning to high levels	**Matrix**: Serum **Method**: Untargeted metabolomics via GC/MS and UPLC-MS/MS; Metabolon Inc.	Repeated measures ANOVA; network and enrichment analysis; false discovery rate adjustment (q-values)	During Olympics → ↓ Air pollution → ↓ Biomarkers (lipid and peptide pathways) After Olympics → ↑ Air pollution → ↑ Biomarkers

Abbreviations: ↑: increase, ↓: decrease, →: indicate. O_3_: Ozone, NO_2_: nitrogen dioxide, PM_2.5_: Inhalable Particulate matter with 2.5 microns or less in diameter, PM_10_: Inhalable Particulate matter with a diameter of 10 microns or less, CO: carbon monoxide, SO_2_: Sulphur dioxide, BC: Black carbon, PM_1_: Particulate matter 1, PM_7_:Particulate matter 7, TSP: Total suspended Particulate matter, EC: Elemental Carbon, TDMPS: Twin Differential Mobility Particle Sizer, SBP: systolic blood pressure, DBP: diastolic blood pressure, PP: pulse pressure, MAP: Mean Arterial Pressure, 12-HETE: 12-hydroxyeicosatetraenoic acid, GST: Glutathione S-transferases, GPx: glutathione peroxidase, GR: glutathione reductase, GRO-α: Growth-Regulated Oncogene alpha, IL-8: Interleukin-8, IP-10: Interferon-gamma-induced Protein-10, MCP-1: Monocyte Chemoattractant Protein-1, RANTES: Regulated upon Activation Normal T cell Expressed and Secreted, MCP-2: Monocyte Chemoattractant Protein-2, TARC: Thymus and Activation-Regulated Chemokine, TAS: Total Antioxidant Status, CD40L: Soluble CD40 Ligand, TNF-α: Tumor Necrosis Factor alpha, CRP: C-Reactive Protein, ICAM-1: Intercellular Adhesion Molecule-1, P-selectin: Platelet Selectin, VCAM-1: Vascular Cell Adhesion Molecule-1, IL-1β: Interleukin-1 beta, EBC: Exhaled Breath Condensate, eNO: Exhaled Nitric Oxide, vWF: von Willebrand Factor, sCD62p: Soluble CD62P, 8-OHdG: 8-Hydroxy-2′-deoxyguanosine, HR: Heart Rate, FeNO: Fractional Exhaled Nitric Oxide, EBC: Exhaled Breath Condensate, MDA: Malondialdehyde, SDNN: Standard Deviation of Normal-to-Normal Intervals, LF power: Low-Frequency Power, HF power: High-Frequency Power, PUFAs: Polyunsaturated Fatty Acids, LCFAs: Long-Chain Fatty Acids, SAH: S-Adenosylhomocysteine, HPLC: high-performance liquid chromatography, UV: ultraviolet, ECD: electron capture detector, ELISA: enzyme-linked immunosorbent assays, GEE: Generalized estimating equations, HR: Heart rate, HRV: heart rate variability, ECD: electrochemical detection, CRP: C-reactive protein, QIC: independence model criterion, GC/MS: Gas chromatography- mass spectrometry, UPLC-MS/MS: Ultra performance liquid chromatography tandem mass spectrometry, LME: Linear mixed-effects.

## Data Availability

No new data were created or analyzed in this study. Data sharing is not applicable to this article.

## Supplementary Materials

The following supporting information can be downloaded at: https://www.mdpi.com/article/10.3390/antiox15050627/s1, Table S1: Critical appraisal tool for quantitative studies (adapted from the Hamilton tool).

## Abbreviations

EPHPP	Effective Public Health Practice Project
PM	Particulate Matter
O_3_	Ozone
PM_2.5_	Particulate Matter ≤ 2.5 µm
SO_2_	Sulphur Dioxide
Nox	Nitrogen Oxides
ROS	Reactive Oxygen Species
NO_2_	Nitrogen Dioxide
AQI	Air Quality Index
PM_10_	Particulate Matter ≤ 10 µm
CO	Carbon Monoxide
PNC	Particle Number Concentration
GAM	Generalized Additive Model
CRP	C-reactive protein
IL-6	Interleukin-6
IL8	Interleukin-8
EBC	Exhaled Breath Condensate
TNF-α	Tumor Necrosis Factor-α
MDA	Malondialdehyde
8-OHdG	8-Hydroxy-2′-deoxyguanosine
HRV	Heart Rate Variability
RANTES	Regulated on Activation, Normal T Cell Expressed and Secreted
MCP-2	Monocyte Chemotactic Protein-2
TARC	Thymus-and-Activation-Regulated Chemokine
GRO- α	Growth-Regulated Oncogene α
sCD40L	Soluble CD40 Ligand
IL-1β	Interleukin-1β
VCAM-1	Vascular Cell Adhesion Molecule-1
ICAM	Intercellular Adhesion Molecule (ICAM-1)
FENO	Fractional exhaled Nitric Oxide
vWF	von Willebrand Factor
sCD62P	Soluble P-Selectin (CD62P)
MAPK	Mitogen-Activated Protein Kinase
NF-kB	Nuclear Factor-κB
AP-1	Activator Protein-1
COPD	Chronic Obstructive Pulmonary Disease

## Appendix A

Search strategy

Launch Date: 7 April 2025

**Table A1:** PubMed.

#1	“Air Pollution”[Mesh] OR “Air Pollutants”[Mesh] OR “Vehicle Emissions”[Mesh] OR “Particulate Matter”[Mesh] OR “Carbon Monoxide”[Mesh] OR “Nitrogen Oxides”[Mesh] OR “Sulfur Dioxide”[Mesh] OR “Volatile Organic Compounds”[Mesh] OR “Lead”[Mesh] OR “Ozone”[Mesh]	371,818
#2	“air pollution”[tiab:~2] OR “air pollutant”[tiab:~2] OR “air pollutants”[tiab:~2] OR “air polluted”[tiab:~2] OR “airborne pollution”[tiab:~2] OR “airborne pollutant”[tiab:~2] OR “airborne pollutants”[tiab:~2] OR “airborne polluted”[tiab:~2] OR “air contamination”[tiab:~2] OR “airborne contamination”[tiab:~2] OR “air contaminant”[tiab:~2] OR “air contaminants”[tiab:~2] OR “airborne contaminant”[tiab:~2] OR “airborne contaminants”[tiab:~2] OR “atmospheric pollution”[tiab:~2] OR “atmospheric pollutant”[tiab:~2] OR “atmospheric pollutants”[tiab:~2] OR “atmospheric polluted”[tiab:~2] OR “atmospheric contamination”[tiab:~2] OR “atmospheric contaminant”[tiab:~2] OR “atmospheric contaminants”[tiab:~2] OR “atmosphere pollution”[tiab:~2] OR “atmosphere pollutant”[tiab:~2] OR “atmosphere pollutants”[tiab:~2] OR “atmosphere polluted”[tiab:~2] OR “atmosphere contamination”[tiab:~2] OR “atmosphere contaminant”[tiab:~2] OR “atmosphere contaminants”[tiab:~2] OR “air quality”[tiab]	73,605
#3	smog[tiab] OR emissions[tiab] OR fumes[tiab] OR “black carbon”[tiab] OR “soot”[tiab] OR “diesel exhaust*”[tiab] OR particulate[tiab] OR “ultrafine particle*”[tiab] OR “ultra-fine particle*”[tiab] OR “PM 2.5”[tiab] OR “PM_2.5_”[tiab] OR “PM 10”[tiab] OR PM10[tiab] OR “PM 0.1”[tiab] OR “PM0.1”[tiab] OR “PM 1”[tiab] OR PM1[tiab] OR “atmospheric particle*”[tiab] OR “carbon monoxide”[tiab] OR “oxide of nitrogen”[tiab] OR “oxides of nitrogen”[tiab] OR “nitrogen oxide*”[tiab] OR “nitrogen dioxide*”[tiab] OR “sulfur dioxide”[tiab] OR “sulfurous anhydride”[tiab] OR “sulphur dioxide”[tiab] OR “sulphurous anhydride”[tiab] OR “volatile organic compound*”[tiab] OR lead[tiab] OR ozone[tiab]	1,097,212
#4	#1 OR #2 OR #3	1,346,829
#5	“Mass Gatherings”[Mesh] OR “Anniversaries and Special Events”[Mesh] OR “Holidays”[Mesh]	11,265
#6	“mass gathering”[tiab:~2] OR “mass gatherings”[tiab:~2] OR “mega-gathering*”[tiab] OR “mega audience”[tiab:~2] OR “mega audiences”[tiab:~2] OR “massive audience”[tiab:~2] OR “massive audiences”[tiab:~2] OR mega-event*[tiab] OR “major event*”[tiab] OR “large-scale event”[tiab:~1] OR “mass event”[tiab:~1] OR “crowd event”[tiab:~1] OR “gathering event”[tiab:~1] OR “high-attendance event”[tiab:~1] OR “international event”[tiab:~1] OR “global event”[tiab:~1] OR “landmark event”[tiab:~1] OR “hallmark event”[tiab:~1] OR “regional event”[tiab:~1] OR “world-class event”[tiab:~2] OR “blockbuster event”[tiab:~2] OR “large-scale events”[tiab:~1] OR “mass events”[tiab:~1] OR “crowd events”[tiab:~1] OR “gathering events”[tiab:~1] OR “high-attendance events”[tiab:~1] OR “international events”[tiab:~1] OR “global events”[tiab:~1] OR “landmark events”[tiab:~1] OR “hallmark events”[tiab:~1] OR “regional events”[tiab:~1] OR “world-class events”[tiab:~2] OR “blockbuster events”[tiab:~2] OR “large crowd”[tiab:~1] OR “large gathering”[tiab:~1] OR “large crowds”[tiab:~1] OR “large gatherings”[tiab:~1] OR “dense crowd”[tiab:~1] OR “dense gathering”[tiab:~1] OR “dense crowds”[tiab:~1] OR “dense gatherings”[tiab:~1] OR “crowd gathering”[tiab:~1] OR “crowds gatherings”[tiab:~1] OR “public congregation”[tiab:~1] OR “public congregations”[tiab:~1] OR festival*[tiab] OR celebration*[tiab] OR holiday*[tiab]	21,690
#7	Hajj[tiab] OR “Kumbh Mela”[tiab] OR “religious gathering*”[tiab] OR pilgrimage*[tiab] OR Carnival[tiab] OR “sporting event*”[tiab] OR “sports event*”[tiab] OR “sport event*”[tiab] OR “international competition*”[tiab] OR Championship*[tiab] OR Games[tiab] OR Olympic[tiab] OR Olympics[tiab] OR Paralympic*[tiab] OR Paraolympic*[tiab] OR “World Cup”[tiab] OR “Grand Prix”[tiab] OR Finals[tiab] OR “World Series”[tiab] OR “Super Bowl”[tiab] OR “Grand Slam”[tiab] OR (Wimbledon[tiab] OR “Roland Garros”[tiab] OR “French Open”[tiab] OR “US Open”[tiab] OR “Australian Open”[tiab] AND tennis[tiab]) OR (“concert”[tiab] NOT “in concert”[tiab]) OR concerts[tiab] OR Expo[tiab] OR Expos[tiab] OR “World Exposition*”[tiab] OR “World’s fair*”[tiab] OR “Universal exhibition”[tiab:~1] OR “Universal exhibitions”[tiab:~1]	36,925
#8	#5 OR #6 OR #7	66,146
#9	“Respiratory Tract Diseases”[Mesh] OR “Cardiovascular Diseases”[Mesh] OR “Hospitalization”[Mesh] OR “Inpatients”[Mesh]	4,736,552
#10	respirat*[tiab] OR pulmon*[tiab] OR bronch*[tiab] OR lung[tiab] OR lungs[tiab] OR airway*[tiab] OR asthma*[tiab] OR laryng*[tiab] OR pneumo*[tiab] OR cardio*[tiab] OR cardia*[tiab] OR heart[tiab] OR myocard*[tiab] OR pericard*[tiab] OR ventricul*[tiab] OR coronar*[tiab] OR vascul*[tiab] OR circulat*[tiab] OR thrombo*[tiab] OR vessel*[tiab] OR vein[tiab] OR veins[tiab] OR arter*[tiab] OR hospital*[tiab] OR admission*[tiab] OR inpatient*[tiab]	7,781,154
#11	“Biomarkers”[Mesh:NoExp] OR “Fractional Exhaled Nitric Oxide Testing”[Mesh] OR “Respiratory Function Tests”[Mesh] OR “Peak Expiratory Flow Rate”[Mesh] OR “Oxidative Stress”[Mesh] OR “Malondialdehyde”[Mesh] OR “Thiobarbituric Acid Reactive Substances”[Mesh] OR “Isoprostanes”[Mesh] OR “Glutathione”[Mesh] OR “8-Hydroxy-2′-Deoxyguanosine”[Mesh] OR “C-Reactive Protein”[Mesh] OR “Interleukins”[Mesh] OR “Pulmonary Surfactant-Associated Protein D”[Mesh] OR “Tumor Necrosis Factor-alpha”[Mesh] OR “Intercellular Adhesion Molecule-1”[Mesh] OR “Intercellular Adhesion Molecule-3”[Mesh] OR “Vascular Cell Adhesion Molecule-1”[Mesh]	1,259,700
#12	biomarker*[tiab] OR marker*[tiab] OR “fractional exhaled nitric oxide”[tiab] OR FENO[tiab] OR spirometr*[tiab] OR “forced expiratory”[tiab] OR “forced expiration”[tiab] OR FEVt[tiab] OR FEV1[tiab] OR “FEV 1”[tiab] OR “forced vital capacit*”[tiab] OR “timed vital capacit*”[tiab] OR FVC[tiab] OR “FEV1/FVC”[tiab] OR “peak expiratory flow”[tiab] OR “peak expiration flow”[tiab] OR “oxidative stress”[tiab] OR “oxidative damage”[tiab] OR “oxidative DNA damage”[tiab] OR “oxidative injur*”[tiab] OR “antioxidative stress”[tiab] OR “oxidant stress”[tiab] OR malondialdehyde[tiab] OR malonylaldehyde[tiab] OR malonaldehyde[tiab] OR malonyldialdehyde[tiab] OR MDA[tiab] OR “lipid peroxidation marker*”[tiab] OR “lipid peroxidation biomarker*”[tiab] OR “thiobarbituric acid reactive substance*”[tiab] OR TBARs[tiab] OR isoprost*[tiab] OR “total antioxidant power”[tiab] OR “total anti-oxidant power”[tiab] OR “total antioxidant capacity”[tiab] OR “total anti-oxidant capacity”[tiab] OR TAOC[tiab] OR T-AOC[tiab] OR glutathion*[tiab] OR GSH[tiab] OR 8-hydroxy-2′-deoxyguanosine[tiab] OR 8-hydroxy-deoxyguanosine[tiab] OR 8-hydroxydeoxyguanosine[tiab] OR 8-hydroxyguanine[tiab] OR 8-hydroxy-guanine[tiab] OR 8-Oxo-2′-Deoxyguanosine[tiab] OR 8-Oxo-Deoxyguanosine[tiab] OR 8-oxo-dGuo[tiab] OR 8-Ohdg[tiab] OR 8OHdG[tiab] OR 8-OH-dG[tiab] OR 8-ohg[tiab] OR 8-hydroxy-g[tiab] OR 8-hydroxy-dg[tiab] OR 8-oxodG[tiab] OR 8-oxodGuo[tiab] OR 8-oxo-dG[tiab] OR 8-OH-2dG*[tiab] OR “C-reactive protein”[tiab] OR CRP[tiab] OR hsCRP[tiab] OR hs-CRP[tiab] OR “high-sensitivity CRP”[tiab] OR interleukin*[tiab] OR IL-6[tiab] OR IL6[tiab] OR IL-1-B[tiab] OR IL-1-beta[tiab] OR IL1-B[tiab] OR IL1-beta[tiab] OR IL-8[tiab] OR IL8[tiab] OR IL10[tiab] OR IL-10[tiab] OR “Krebs von den Lungen”[tiab] OR KL-6[tiab] OR “surfactant-associated protein D”[tiab] OR “surfactant protein D”[tiab] OR “surfactant-associated glycoprotein D”[tiab] OR SP-D[tiab] OR “Tumor Necrosis Factor-alpha”[tiab] OR TNF-alpha[tiab] OR TNF-a[tiab] OR “Neutrophil-to-Lymphocyte Ratio”[tiab] OR “Neutrophil Lymphocyte Ratio”[tiab] OR “Neutrophil/Lymphocyte Ratio”[tiab] OR NLR[tiab] OR “intercellular adhesion molecule”[tiab] OR “intercellular cell adhesion molecule”[tiab] OR “inter-cellular adhesion molecule”[tiab] OR “inter-cellular cell adhesion molecule”[tiab:~0] OR I-CAM*[tiab] OR ICAM*[tiab] OR “vascular cell adhesion molecule”[tiab] OR “vascular adhesion molecule”[tiab] OR V-CAM*[tiab] OR VCAM*[tiab]	2,459,012
#13	#9 OR #10 OR #11 OR #12	11,016,563
#14	#4 AND #8 AND #13	1074

**Table A2:** Embase (on Embase.com).

#1	‘air pollution’/exp OR ‘air pollutant’/exp OR ‘exhaust gas’/exp OR ‘particulate matter’/exp OR ‘carbon monoxide’/exp OR ‘nitrogen oxide’/exp OR ‘sulfur dioxide’/exp OR ‘volatile organic compound’/exp OR ‘lead’/exp OR ‘ozone’/exp	438,521
#2	(air NEAR/3 pollut*):ti,ab,kw OR (airborne NEAR/3 pollut*):ti,ab,kw OR (air NEAR/3 contamina*):ti,ab,kw OR (airborne NEAR/3 contamina*):ti,ab,kw OR (atmospher* NEAR/3 pollut*):ti,ab,kw OR (atmospher* NEAR/2 contamina*):ti,ab,kw OR ‘air quality’:ti,ab,kw	98,616
#3	smog:ti,ab,kw OR emissions:ti,ab,kw OR fumes:ti,ab,kw OR ‘black carbon’:ti,ab,kw OR soot:ti,ab,kw OR ‘diesel exhaust*’:ti,ab,kw OR particulate:ti,ab,kw OR ‘ultrafine particle*’:ti,ab,kw OR ‘ultra-fine particle*’:ti,ab,kw OR ‘PM 2.5’:ti,ab,kw OR ‘PM_2.5_’:ti,ab,kw OR ‘PM 10’:ti,ab,kw OR PM10:ti,ab,kw OR ‘PM 0.1’:ti,ab,kw OR ‘PM0.1’:ti,ab,kw OR ‘PM 1’:ti,ab,kw OR PM1:ti,ab,kw OR ‘atmospheric particle*’:ti,ab,kw OR ‘carbon monoxide’:ti,ab,kw OR ‘oxide of nitrogen’:ti,ab,kw OR ‘oxides of nitrogen’:ti,ab,kw OR ‘nitrogen oxide*’:ti,ab,kw OR ‘nitrogen dioxide*’:ti,ab,kw OR ‘sulfur dioxide’:ti,ab,kw OR ‘sulfurous anhydride’:ti,ab,kw OR ‘sulphur dioxide’:ti,ab,kw OR ‘sulphurous anhydride’:ti,ab,kw OR ‘volatile organic compound*’:ti,ab,kw OR lead:ti,ab,kw OR ozone:ti,ab,kw	1,469,016
#4	#1 OR #2 OR #3	1,682,514
#5	‘mass gathering’/exp	460
#6	(mass NEAR/3 gathering*):ti,ab,kw OR ‘mega-gathering*’:ti,ab,kw OR ((mega OR massive) NEAR/3 audience*):ti,ab,kw OR ‘mega-event*’:ti,ab,kw OR ‘major event*’:ti,ab,kw OR ((large-scale OR mass OR crowd OR gathering OR high-attendance OR international OR global OR landmark OR hallmark OR regional OR world-class OR blockbuster) NEAR/2 event*):ti,ab,kw OR ((large OR dense) NEAR/2 (crowd* OR gathering*)):ti,ab,kw OR (crowd* NEAR/2 gathering*):ti,ab,kw OR (public NEAR/2 congregation*):ti,ab,kw OR festival*:ti,ab,kw OR celebration*:ti,ab,kw OR holiday*:ti,ab,kw	29,208
#7	Hajj:ti,ab,kw OR ‘Kumbh Mela’:ti,ab,kw OR ‘religious gathering*’:ti,ab,kw OR pilgrimage*:ti,ab,kw OR Carnival:ti,ab,kw OR ‘sporting event*’:ti,ab,kw OR ‘sports event*’:ti,ab,kw OR ‘sport event*’:ti,ab,kw OR ‘international competition*’:ti,ab,kw OR Championship*:ti,ab,kw OR Games:ti,ab,kw OR Oylimpic:ti,ab,kw OR Olympics:ti,ab,kw OR Paralympic*:ti,ab,kw OR Paraolympic*:ti,ab,kw OR ‘World Cup’:ti,ab,kw OR ‘Grand Prix’:ti,ab,kw OR Finals:ti,ab,kw OR ‘World Series’:ti,ab,kw OR ‘Super Bowl’:ti,ab,kw OR ‘Grand Slam’:ti,ab,kw OR (Wimbledon:ti,ab,kw OR ‘Roland Garros’:ti,ab,kw OR ‘French Open’:ti,ab,kw OR ‘US Open’:ti,ab,kw OR ‘Australian Open’:ti,ab,kw AND tennis:ti,ab,kw) OR (concert:ti,ab,kw NOT ‘in concert’:ti,ab,kw) OR concerts:ti,ab,kw OR Expo:ti,ab,kw OR Expos:ti,ab,kw OR ‘World Exposition’:ti,ab,kw OR (Universal NEAR/2 exhibition*):ti,ab,kw OR ‘World s fair*’:ti,ab,kw	40,503
#8	#5 OR #6 OR #7	68,510
#9	‘respiratory tract disease’/exp OR ‘cardiovascular disease’/exp OR ‘hospitalization’/exp OR ‘hospital patient’/exp	8,998,595
#10	respirat*:ti,ab,kw OR pulmon*:ti,ab,kw OR bronch*:ti,ab,kw OR lung:ti,ab,kw OR lungs:ti,ab,kw OR airway*:ti,ab,kw OR asthma*:ti,ab,kw OR laryng*:ti,ab,kw OR pneumo*:ti,ab,kw OR cardio*:ti,ab,kw OR cardia*:ti,ab,kw OR heart:ti,ab,kw OR myocard*:ti,ab,kw OR pericard*:ti,ab,kw OR ventricul*:ti,ab,kw OR coronar*:ti,ab,kw OR vascul*:ti,ab,kw OR circulat*:ti,ab,kw OR thrombo*:ti,ab,kw OR vessel*:ti,ab,kw OR vein:ti,ab,kw OR veins:ti,ab,kw OR arter*:ti,ab,kw OR hospital*:ti,ab,kw OR admission*:ti,ab,kw OR inpatient*:ti,ab,kw	10,899,533
#11	‘biological marker’/de OR ‘fractional exhaled nitric oxide test’/exp OR ‘fractional exhaled nitric oxide’/exp OR ‘lung function test’/exp OR ‘forced expiratory volume’/exp OR ‘forced vital capacity’/exp OR ‘oxidative stress’/exp OR ‘malonaldehyde’/exp OR ‘thiobarbituric acid reactive substance’/exp OR ‘isoprostane derivative’/exp OR ‘total antioxidant capacity’/exp OR ‘glutathione’/exp OR ‘8 hydroxydeoxyguanosine’/exp OR ‘C reactive protein’/exp OR ‘interleukin derivative’/exp OR ‘interleukin 1beta’/exp OR ‘interleukin 10’/exp OR ‘krebs von den lungen 6’/exp OR ‘surfactant protein D’/exp OR ‘tumor necrosis factor’/exp OR ‘neutrophil lymphocyte ratio’/exp OR ‘intercellular adhesion molecule’/exp OR ‘intercellular adhesion molecule 1’/exp OR ‘intercellular adhesion molecule 2’/exp OR ‘intercellular adhesion molecule 3’/exp OR ‘vascular cell adhesion molecule’/exp OR ‘vascular cell adhesion molecule 1’/exp	2,219,996
#12	biomarker*:ti,ab,kw OR marker*:ti,ab,kw OR ‘fractional exhaled nitric oxide’:ti,ab,kw OR FENO:ti,ab,kw OR spirometr*:ti,ab,kw OR ‘forced expiratory’:ti,ab,kw OR ‘forced expiration’:ti,ab,kw OR FEVt:ti,ab,kw OR FEV1:ti,ab,kw OR ‘FEV 1’:ti,ab,kw OR ‘forced vital capacit*’:ti,ab,kw OR ‘timed vital capacit*’:ti,ab,kw OR FVC:ti,ab,kw OR ‘FEV1/FVC’:ti,ab,kw OR ‘peak expiratory flow’:ti,ab,kw OR ‘peak expiration flow’:ti,ab,kw OR ‘oxidative stress’:ti,ab,kw OR ‘oxidative damage’:ti,ab,kw OR ‘oxidative DNA damage’:ti,ab,kw OR ‘oxidative injur*’:ti,ab,kw OR ‘antioxidative stress’:ti,ab,kw OR ‘oxidant stress’:ti,ab,kw OR malondialdehyde:ti,ab,kw OR malonylaldehyde:ti,ab,kw OR malonaldehyde:ti,ab,kw OR malonyldialdehyde:ti,ab,kw OR MDA:ti,ab,kw OR ‘lipid peroxidation marker*’:ti,ab,kw OR ‘lipid peroxidation biomarker*’:ti,ab,kw OR ‘thiobarbituric acid reactive substance*’:ti,ab,kw OR TBARs:ti,ab,kw OR isoprost*:ti,ab,kw OR ‘total antioxidant power’:ti,ab,kw OR ‘total anti-oxidant power’:ti,ab,kw OR ‘total antioxidant capacity’:ti,ab,kw OR ‘total anti-oxidant capacity’:ti,ab,kw OR TAOC:ti,ab,kw OR T-AOC:ti,ab,kw OR glutathion*:ti,ab,kw OR GSH:ti,ab,kw OR 8-hydroxy-2-deoxyguanosine:ti,ab,kw OR 8-hydroxy-deoxyguanosine:ti,ab,kw OR 8-hydroxydeoxyguanosine:ti,ab,kw OR 8-hydroxyguanine:ti,ab,kw OR 8-hydroxy-guanine:ti,ab,kw OR 8-Oxo-2-Deoxyguanosine:ti,ab,kw OR 8-Oxo-Deoxyguanosine:ti,ab,kw OR 8-oxo-dGuo:ti,ab,kw OR 8-Ohdg:ti,ab,kw OR 8OHdG:ti,ab,kw OR 8-OH-dG:ti,ab,kw OR 8-ohg:ti,ab,kw OR 8-hydroxy-g:ti,ab,kw OR 8-hydroxy-dg:ti,ab,kw OR 8-oxodG:ti,ab,kw OR 8-oxodGuo:ti,ab,kw OR 8-oxo-dG:ti,ab,kw OR 8-OH-2dG*:ti,ab,kw OR ‘C-reactive protein’:ti,ab,kw OR CRP:ti,ab,kw OR hsCRP:ti,ab,kw OR hs-CRP:ti,ab,kw OR ‘high-sensitivity CRP’:ti,ab,kw OR interleukin*:ti,ab,kw OR IL-6:ti,ab,kw OR IL6:ti,ab,kw OR IL-1-B:ti,ab,kw OR IL-1-beta:ti,ab,kw OR IL1-B:ti,ab,kw OR IL1-beta:ti,ab,kw OR IL-8:ti,ab,kw OR IL8:ti,ab,kw OR IL10:ti,ab,kw OR IL-10:ti,ab,kw OR ‘Krebs von den Lungen’:ti,ab,kw OR KL-6:ti,ab,kw OR ‘surfactant-associated protein D’:ti,ab,kw OR ‘surfactant protein D’:ti,ab,kw OR ‘surfactant-associated glycoprotein D’:ti,ab,kw OR SP-D:ti,ab,kw OR ‘Tumor Necrosis Factor-alpha’:ti,ab,kw OR TNF-alpha:ti,ab,kw OR TNF-a:ti,ab,kw OR ‘Neutrophil-to-Lymphocyte Ratio’:ti,ab,kw OR ‘Neutrophil Lymphocyte Ratio’:ti,ab,kw OR ‘Neutrophil/Lymphocyte Ratio’:ti,ab,kw OR NLR:ti,ab,kw OR ‘intercellular adhesion molecule’:ti,ab,kw OR ‘intercellular cell adhesion molecule’:ti,ab,kw OR ‘inter-cellular adhesion molecule’:ti,ab,kw OR ‘inter-cellular cell adhesion molecule’:ti,ab,kw OR I-CAM*:ti,ab,kw OR ICAM*:ti,ab,kw OR ‘vascular cell adhesion molecule’:ti,ab,kw OR ‘vascular adhesion molecule’:ti,ab,kw OR V-CAM*:ti,ab,kw OR VCAM*:ti,ab,kw	3,355,859
#13	#9 OR #10 OR #11 OR #12	15,933,794
#14	#4 AND #8 AND #13	1583

**Table A3:** Scopus.

1	TITLE-ABS-KEY ((air W/2 pollut*) OR (airborne W/2 pollut*) OR (air W/2 contamina*) OR (airborne W/2 contamina*) OR (atmospher* W/2 pollut*) OR (atmospher* W/2 contamina*) OR “air quality” OR “smog” OR {emissions} OR {fumes} OR “black carbon” OR “soot” OR “diesel exhaust*” OR “particulate” OR “ultrafine particle*” OR “ultra-fine particle*” OR “PM 2.5” OR “PM_2.5_” OR “PM 10” OR “PM10” OR “PM 0.1” OR “PM0.1” OR “PM 1” OR “PM1” OR “atmospheric particle*” OR “carbon monoxide” OR “oxide of nitrogen” OR “oxides of nitrogen” OR “nitrogen oxide*” OR “nitrogen dioxide*” OR “sulfur dioxide” OR “sulfurous anhydride” OR “sulphur dioxide” OR “sulphurous anhydride” OR “volatile organic compound*” OR lead OR ozone)	5,308,908
2	TITLE-ABS-KEY ((mass W/2 gathering*) OR “mega-gathering*” OR ((mega OR massive) W/2 audience*) OR “mega-event*” OR “major event*” OR ((large-scale OR mass OR crowd OR gathering OR high-attendance OR international OR global OR landmark OR hallmark OR regional OR world-class OR blockbuster) W/1 event*) OR ((large OR dense) W/1 (crowd* OR gathering*)) OR (crowd* W/1 gathering*) OR (public W/1 congregation*) OR festival* OR celebration* OR holiday* OR Hajj OR “Kumbh Mela” OR “religious gathering*” OR pilgrimage* OR Carnival OR “sporting event*” OR “sports event*” OR “sport event*” OR “international competition*” OR Championship* OR {Games} OR Oylimpic OR Olympics OR Paralympic* OR Paraolympic* OR “World Cup” OR “Grand Prix” OR {Finals} OR “World Series” OR “Super Bowl” OR “Grand Slam” OR (Wimbledon OR “Roland Garros” OR “French Open” OR “US Open” OR “Australian Open” AND tennis) OR (concert AND NOT “in concert”) OR {concerts} OR Expo OR Expos OR “World Exposition” OR (universal W/1 exhibition*) OR “World’s fair*”)	389,761
3	TITLE-ABS-KEY (respirat* OR pulmon* OR bronch* OR lung OR lungs OR airway* OR asthma* OR laryng* OR pneumo* OR cardio* OR cardia* OR heart OR myocard* OR pericard* OR ventricul* OR coronar* OR vascul* OR circulat* OR thrombo* OR vessel* OR vein OR veins OR arter* OR hospital* OR admission* OR inpatient* OR biomarker* OR marker* OR “fractional exhaled nitric oxide” OR “FENO” OR spirometr* OR “forced expiratory” OR “forced expiration” OR “FEVt” OR “FEV1” OR “FEV 1” OR “forced vital capacit*” OR “timed vital capacit*” OR “FVC” OR “FEV1/FVC” OR “peak expiratory flow” OR “peak expiration flow” OR “oxidative stress” OR “oxidative damage” OR “oxidative DNA damage” OR “oxidative injur*” OR “antioxidative stress” OR “oxidant stress” OR malondialdehyde OR malonylaldehyde OR malonaldehyde OR malonyldialdehyde OR “MDA” OR “lipid peroxidation marker*” OR “lipid peroxidation biomarker*” OR “thiobarbituric acid reactive substance*” OR “TBARs” OR isoprost* OR “total antioxidant power” OR “total anti-oxidant power” OR “total antioxidant capacity” OR “total anti-oxidant capacity” OR “TAOC” OR “T-AOC” OR glutathion* OR “GSH” OR “8-hydroxy-2-deoxyguanosine” OR “8-hydroxy-deoxyguanosine” OR “8-hydroxydeoxyguanosine” OR “8-hydroxyguanine” OR “8-hydroxy-guanine” OR “8-Oxo-2-Deoxyguanosine” OR “8-Oxo-Deoxyguanosine” OR “8-oxo-dGuo” OR “8-Ohdg” OR 8ohdg OR “8-OH-dG” OR “8-ohg” OR “8-hydroxy-g” OR “8-hydroxy-dg” OR “8-oxodG” OR “8-oxodGuo” OR “8-oxo-dG” OR “8-OH-2dG*” OR “C-reactive protein” OR “CRP” OR hscrp OR “hs-CRP” OR “high-sensitivity CRP” OR interleukin* OR “IL-6” OR “IL6” OR “IL-1-B” OR “IL-1-beta” OR “IL1-B” OR “IL1-beta” OR “IL-8” OR “IL8” OR “IL10” OR “IL-10” OR “Krebs von den Lungen” OR “KL-6” OR “surfactant-associated protein D” OR “surfactant protein D” OR “surfactant-associated glycoprotein D” OR “SP-D” OR “Tumor Necrosis Factor-alpha” OR “TNF-alpha” OR “TNF-a” OR “Neutrophil-to-Lymphocyte Ratio” OR “Neutrophil Lymphocyte Ratio” OR “Neutrophil/Lymphocyte Ratio” OR “NLR” OR “intercellular adhesion molecule” OR “intercellular cell adhesion molecule” OR “inter-cellular adhesion molecule” OR “inter-cellular cell adhesion molecule” OR “I-CAM*” OR ICAM* OR “vascular cell adhesion molecule” OR “vascular adhesion molecule” OR “V-CAM*” OR VCAM*)	15,429,231
4	1 AND 2 AND 3	2121

**Table A4:** Web of Science.

1	TS = ((air NEAR/2 pollut*) OR (airborne NEAR/2 pollut*) OR (air NEAR/2 contamina*) OR (airborne NEAR/2 contamina*) OR (atmospher* NEAR/2 pollut*) OR (atmospher* NEAR/2 contamina*) OR “air quality” OR smog OR emissions OR fumes OR “black carbon” OR soot OR “diesel exhaust*” OR particulate OR “ultrafine particle*” OR “ultra-fine particle*” OR “PM 2.5” OR “PM_2.5_” OR “PM 10” OR PM10 OR “PM 0.1” OR “PM0.1” OR PM 1 OR PM1 OR “atmospheric particle*” OR “carbon monoxide” OR “oxide of nitrogen” OR “oxides of nitrogen” OR “nitrogen oxide*” OR “nitrogen dioxide*” OR “sulfur dioxide” OR “sulfurous anhydride” OR “sulphur dioxide” OR “sulphurous anhydride” OR “volatile organic compound*” OR lead OR ozone)	2,713,334
2	TS = ((mass NEAR/2 gathering*) OR “mega-gathering*” OR ((mega OR massive) NEAR/2 audience*) OR “mega-event*” OR “major event*” OR ((large-scale OR mass OR crowd OR gathering OR high-attendance OR international OR global OR landmark OR hallmark OR regional OR world-class OR blockbuster) NEAR/1 event*) OR ((large OR dense) NEAR/1 (crowd* OR gathering*)) OR (crowd* NEAR/1 gathering*) OR (public NEAR/1 congregation*) OR festival* OR celebration* OR holiday* OR Hajj OR “Kumbh Mela” OR “religious gathering*” OR pilgrimage* OR Carnival OR “sporting event*” OR “sports event*” OR “sport event*” OR “international competition*” OR Championship* OR Games OR Oylimpic OR Olympics OR Paralympic* OR Paraolympic* OR “World Cup” OR “Grand Prix” OR Finals OR “World Series” OR “Super Bowl” OR “Grand Slam” OR (Wimbledon OR “Roland Garros” OR “French Open” OR “US Open” OR “Australian Open” AND tennis ) OR (concert NOT “in concert”) OR concerts OR Expo OR Expos OR “World Exposition” OR (universal NEAR/1 exhibition*) OR “World’s fair*”)	282,758
3	TS = (respirat* OR pulmon* OR bronch* OR lung OR lungs OR airway* OR asthma* OR laryng* OR pneumo* OR cardio* OR cardia* OR heart OR myocard* OR pericard* OR ventricul* OR coronar* OR vascul* OR circulat* OR thrombo* OR vessel* OR vein OR veins OR arter* OR hospital* OR admission* OR inpatient* OR biomarker* OR marker* OR “fractional exhaled nitric oxide” OR FENO OR spirometr* OR “forced expiratory” OR “forced expiration” OR FEVt OR FEV1 OR “FEV 1” OR “forced vital capacit*” OR “timed vital capacit*” OR FVC OR “FEV1/FVC” OR “peak expiratory flow” OR “peak expiration flow” OR “oxidative stress” OR “oxidative damage” OR “oxidative DNA damage” OR “oxidative injur*” OR “antioxidative stress” OR “oxidant stress” OR malondialdehyde OR malonylaldehyde OR malonaldehyde OR malonyldialdehyde OR MDA OR “lipid peroxidation marker*” OR “lipid peroxidation biomarker*” OR “thiobarbituric acid reactive substance*” OR TBARs OR isoprost* OR “total antioxidant power” OR “total anti-oxidant power” OR “total antioxidant capacity” OR “total anti-oxidant capacity” OR TAOC OR “T-AOC” OR glutathion* OR GSH OR “8-hydroxy-2-deoxyguanosine” OR “8-hydroxy-deoxyguanosine” OR “8-hydroxydeoxyguanosine” OR “8-hydroxyguanine” OR “8-hydroxy-guanine” OR “8-Oxo-2-Deoxyguanosine” OR “8-Oxo-Deoxyguanosine” OR “8-oxo-dGuo” OR “8-Ohdg” OR 8ohdg OR “8-OH-dG” OR “8-ohg” OR “8-hydroxy-g” OR “8-hydroxy-dg” OR “8-oxodG” OR “8-oxodGuo” OR “8-oxo-dG” OR “8-OH-2dG*” OR “C-reactive protein” OR CRP OR hscrp OR “hs-CRP” OR “high-sensitivity CRP” OR interleukin* OR “IL-6” OR IL6 OR “IL-1-B” OR “IL-1-beta” OR “IL1-B” OR “IL1-beta” OR “IL-8” OR IL8 OR IL10 OR “IL-10” OR “Krebs von den Lungen” OR “KL-6” OR “surfactant-associated protein D” OR “surfactant protein D” OR “surfactant-associated glycoprotein D” OR “SP-D” OR “Tumor Necrosis Factor-alpha” OR “TNF-alpha” OR “TNF-a” OR “Neutrophil-to-Lymphocyte Ratio” OR “Neutrophil Lymphocyte Ratio” OR “Neutrophil/Lymphocyte Ratio” OR NLR OR “intercellular adhesion molecule” OR “intercellular cell adhesion molecule” OR “inter-cellular adhesion molecule” OR “inter-cellular cell adhesion molecule” OR “I-CAM*” OR ICAM* OR “vascular cell adhesion molecule” OR “vascular adhesion molecule” OR “V-CAM*” OR VCAM*)	10,830,505
4	1 AND 2 AND 3	1429
